# Differentiation block in acute myeloid leukemia regulated by intronic sequences of *FTO*

**DOI:** 10.1016/j.isci.2023.107319

**Published:** 2023-07-11

**Authors:** Francesco Camera, Isabel Romero-Camarero, Bradley H. Revell, Fabio M.R. Amaral, Oliver J. Sinclair, Fabrizio Simeoni, Daniel H. Wiseman, Lovorka Stojic, Tim C.P. Somervaille

**Affiliations:** 1 Leukaemia Biology Laboratory, Cancer Research UK Manchester Institute, The University of Manchester, The Oglesby Cancer Research Centre Building, 555 Wilmslow Road, M20 4GJ Manchester, UK; 2 Epigenetics of Haematopoiesis Group, Oglesby Cancer Research Building, The University of Manchester, M20 4GJ Manchester, UK; 3 Centre for Cancer Cell and Molecular Biology, Barts Cancer Institute, Queen Mary University of London, Charterhouse Square, EC1M 6BQ London, UK

**Keywords:** Molecular mechanism of gene regulation, Epigenetics, Cancer, Transcriptomics

## Abstract

Iroquois transcription factor gene *IRX3* is highly expressed in 20–30% of acute myeloid leukemia (AML) and contributes to the pathognomonic differentiation block. Intron 8 *FTO* sequences ∼220kB downstream of *IRX3* exhibit histone acetylation, DNA methylation, and contacts with the *IRX3* promoter, which correlate with *IRX3* expression. Deletion of these intronic elements confirms a role in positively regulating *IRX3*. RNAseq revealed long non-coding (lnc) transcripts arising from this locus. *FTO*-lncAML knockdown (KD) induced differentiation of AML cells, loss of clonogenic activity, and reduced *FTO* intron 8:*IRX3* promoter contacts. While both *FTO*-lncAML KD and *IRX3* KD induced differentiation, *FTO*-lncAML but not *IRX3* KD led to HOXA downregulation suggesting transcript activity *in trans*. *FTO*-lncAML^high^ AML samples expressed higher levels of HOXA and lower levels of differentiation genes. Thus, a regulatory module in *FTO* intron 8 consisting of clustered enhancer elements and a long non-coding RNA is active in human AML, impeding myeloid differentiation.

## Highlights

• *FTO* intron 8 contains a regulatory region that controls expression of *IRX3* in AML
• Long non-coding transcripts arise from *FTO* intron 8 in NPM1-mutated AML
• *FTO*-lncAML expression correlates with higher expression of stem cell genes
• *FTO*-lncAML knockdown induces myeloid differentiation in AML

## Graphical abstract

**Figure:**
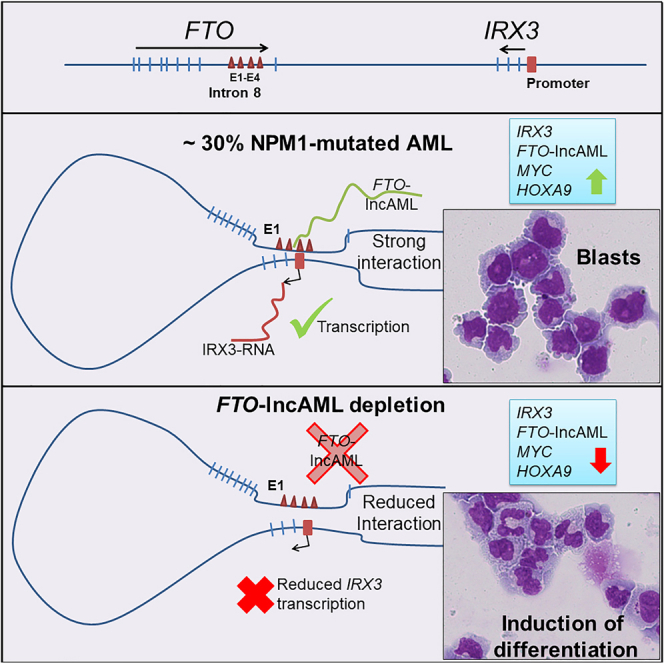

## Introduction

Acute myeloid leukemia (AML) is a genetically heterogeneous group of often proliferative malignancies characterized by a hierarchically organized cellular structure and accumulation of poorly differentiated myeloid blast cells.^1^ Abnormal or impaired differentiation is a key pathologic feature of the disease, and drugs that induce leukemia cell differentiation are already components of effective therapeutic regimens, most notably in acute promyelocytic leukemia.^2^ While normal myeloid differentiation is controlled through the coordinate and balanced expression of critical transcription factors such as *RUNX1*, *CEBPA*, *ETS* factors, *GFI1*, and *IRF8* among others, in leukemia a range of mutations, including balanced translocations, disrupts their proper function. Related to this, we recently reported that transcription factors not normally expressed in myeloid differentiation are frequently mis-expressed to functional effect in AML, to confer or deepen the differentiation block.

One of these is the Iroquois homeodomain transcription factor gene *IRX3* which is highly expressed in the developing nervous system, limb buds, kidney, and heart^3^; and with its paralog *IRX5* makes essential contributions to cardiac and skeletal development.^4^^,^^5^^,^^6^^,^^7^ Importantly, non-coding variation within introns 1 and 2 of *FTO* (for fat mass and obesity-associated) which sits 200–500KB downstream of *IRX3*, provides the strongest genetic association for risk of human obesity. Adult *Irx3*^−/−^ mice have increased basal metabolic rate and reduced fat mass, with browning of white adipose tissue, attributable to loss of hypothalamic^7^ or preadipocyte^8^ *Irx3* expression. A specific variant inside the obesity-associated region (rs1421085 T→C) abrogates the binding of the ARID5B repressor leading to activation of a strong enhancer that promotes the expression of *IRX3* and *IRX5* during adipocyte development.^8^ Interestingly, recent findings indicate that activation of *IRX3* in macrophages may control body weight through promoting transcription of proinflammatory cytokines and repression of adipocyte adrenergic signaling^9^; and transgenerational inheritance of obesity is regulated by activation of *IRX3* in hypothalamic neurons by enhancers localized in intron 8 of *FTO*.^10^

In leukemia, *IRX3* is overexpressed in about 30% of cases of AML, 50% of T-acute lymphoblastic leukemia, and 20% of B-acute lymphoblastic leukemia, while its expression is almost undetectable during normal hematopoiesis and in mature blood cells.^11^ *IRX3* is co-expressed with HOXA genes in primary AML samples, in particular in cases with a normal karyotype or with mutations in *NPM1* and/or *FLT3*. Functional experiments demonstrated that *IRX3* expression alone is sufficient to immortalize murine hematopoietic stem and progenitor cells *in vitro*, and *in vivo* it collaborates with *Hoxa9* to confer a differentiation block in murine AML.^11^ While our prior study indicated that reduced Polycomb repressive activity surrounding *IRX3* contributes to its activation in AML,^11^ the upstream mechanisms underlying this cell-type-inappropriate gene activation remain unclear. To further explore the complexity of *IRX3* regulation in human AML we searched the topologically associating domain (TAD) within which *IRX3* sits for candidate regulatory elements that might control *IRX3* gene expression.

## Results

### The *IRX3* topologically associating domain

To identify the TAD within which *IRX3* is located, we made use of published HiC datasets for THP1 AML cells^12^ (Figure S1A) and multiple primary tissues and cell lines^13^ (Figure S1B). These show that *IRX3* lies within a TAD which also contains two other members of the Iroquois homeobox family (*IRX5* and *IRX6*), the protein-coding genes *FTO* and *RPGRIP1L*, and the long non-coding RNA *CRNDE*. To provide additional confirmation in an independent AML cell line, we performed 4Cseq in Fujioka cells which express *IRX3* at a high level (Figure S1C) using a viewpoint within the *IRX3* promoter and found that the pattern of interactions was restricted to the predicted *IRX3* TAD (Figure S1C).

### Identification of a candidate regulatory region for *IRX3* in intron 8 of *FTO*

**Figure 1:**
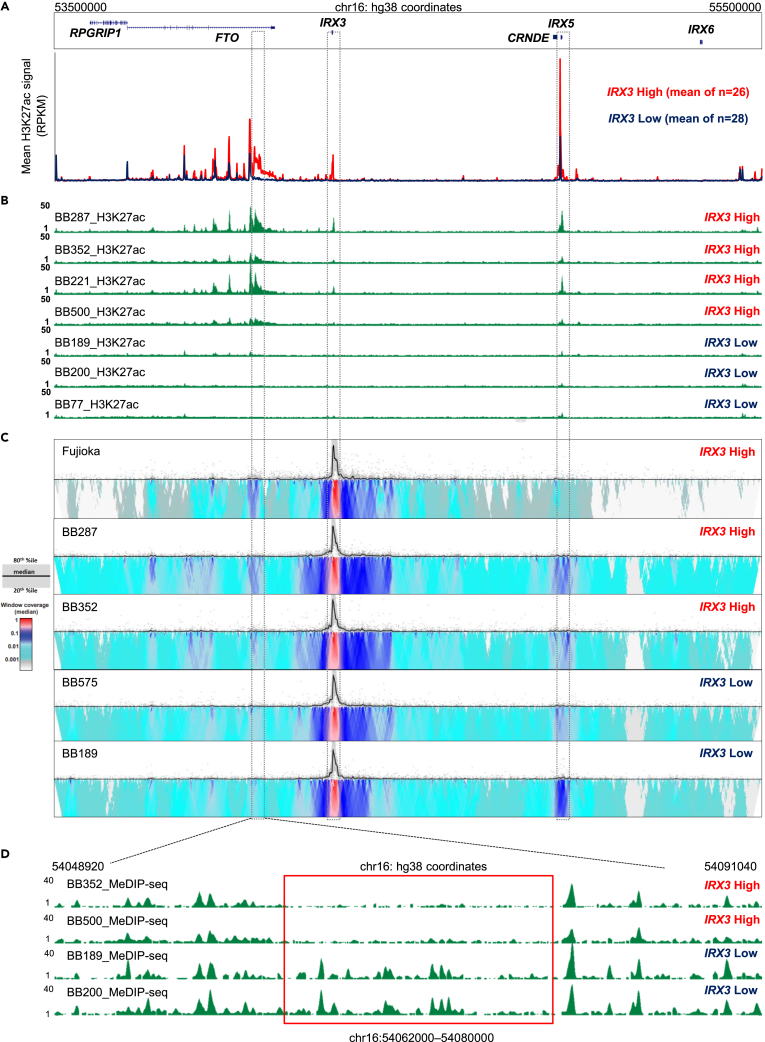
Identification of a candidate regulatory region for *IRX3* in intron 8 of *FTO* (A) Graph shows mean H3K27ac ChIP signal over the *IRX3* TAD for 26 *IRX3*^*high*^ (red) and 28 *IRX3*^*low*^ (blue) primary samples. Data are derived from McKeown et al.^14^ The three areas with the highest differential H3K27ac ChIP signal are indicated by dashed boxes. (B) ChIPseq tracks for H3K27ac across the *IRX3* TAD for the indicated biobank primary AML samples. (C) 4Cseq plots for the indicated cell line and primary AML samples. The strength of the interaction with the *IRX3* promoter is shown as a gradient from red (highest interaction) to blue to cyan to white (lowest interaction). (D) MeDIP-seq tracks for the indicated primary AML samples. The red box highlights the region of differential DNA methylation. See also Figure S1 and Table S1.

To identify regulatory elements that might control the expression of *IRX3* within the TAD, we compared mean H3K27Ac chromatin immunoprecipitation (ChIP) signal in 26 *IRX3*^high^ versus 28 *IRX3*^low^ primary AML samples from a published dataset comprising samples from a range of molecular subtypes^14^ (Figure 1A; Table S1). H3K27ac is a well-established marker of active enhancers. We identified three regions with significantly higher H3K27Ac ChIP signal in *IRX3*^high^ samples (mean fold change ≥4 and p ≤ 0.001). As expected, two of these regions corresponded to the promoters and gene bodies of *IRX3* and *IRX5*; we have previously reported that expression of *IRX3* and *IRX5* correlate one with another in human AML.^11^ Interestingly, the third differentially acetylated region was located inside the last intron of *FTO*, spanning an area of ∼32kb (chr16:54,050,000–54,082,000) at a distance of ∼220kb from the *IRX3* promoter. While this is consistent with prior studies which identified *FTO* introns as the location of regulatory elements controlling expression of *IRX3*,^8^ these were located elsewhere in the gene and an *IRX3* enhancer in this location has not previously been noted.

We confirmed findings from the McKeown et al. dataset^14^ with our own H3K27Ac ChIPseq analysis of seven primary AML samples, all with an *NPM1* mutation (Figure 1B; Table S1). To further evaluate enhancer:promoter contacts we performed additional 4Cseq in a subset of our primary patient AML samples where sufficient material was available, and in Fujioka AML cells (Figure 1C). Consistently, by comparison with the *IRX3*^low^ samples there were stronger contacts in IRX3^high^ samples between the differentially acetylated region localized in the last intron of *FTO* and the *IRX3* promoter. In keeping with the observations for differential acetylation and altered genomic interactions, an analysis of DNA methylation using methylated DNA immunoprecipitation sequencing (MeDIPseq) in two *IRX3*^high^ and two *IRX3*^low^ primary patient samples showed substantially reduced methylation in *IRX3*^high^ versus *IRX3*^low^ cases across the differentially acetylated region (Figure 1D). Taken together, these analyses suggest the presence in both cell lines and primary human AML samples of a candidate *IRX3* regulatory region within the final intron (intron 8) of FTO.

### Elements in intron 8 of *FTO* positively regulates *IRX3* expression in AML

**Figure 2:**
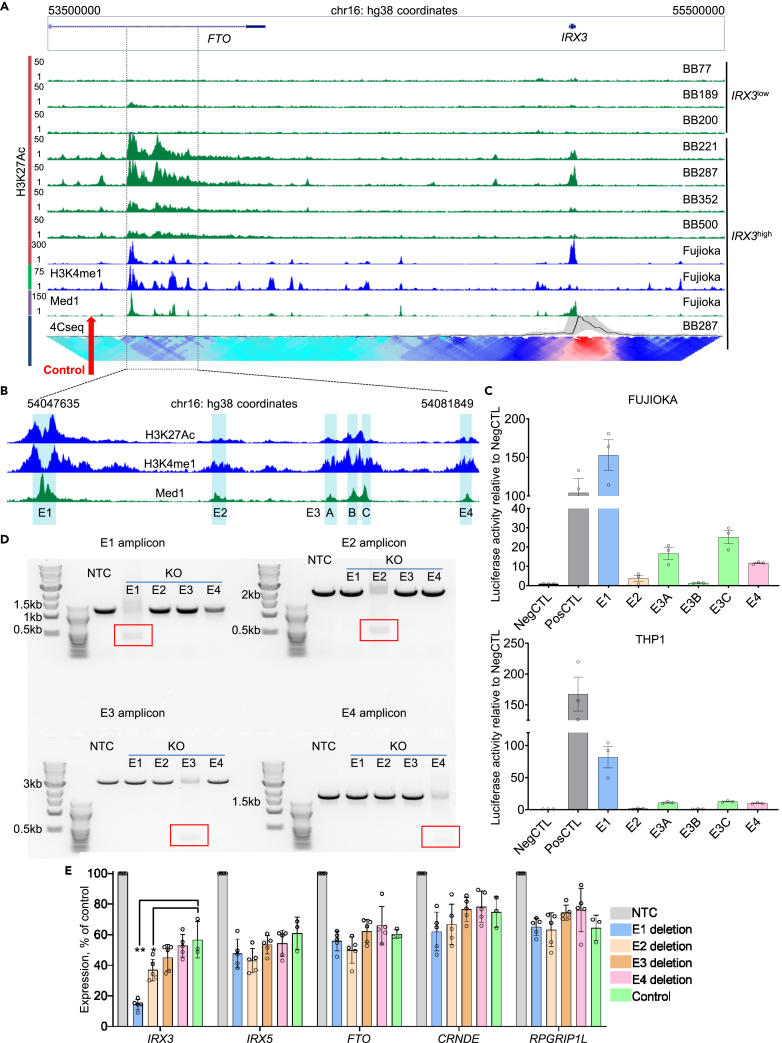
Elements in intron 8 of *FTO* positively regulate *IRX3* expression in AML (A) ChIPseq tracks for the indicated factors in the indicated human AML cells lines and primary samples. The candidate regulatory region is marked by dashed lines. The lower track shows the contact profile generated from 4Cseq in an *IRX3*^high^ primary sample. Red arrow indicates location of region targeted by control CRISPR guides. (B) Enlarged image of candidate regulatory region. The putative regulatory sequences (E1–E4) are marked by blue boxes. (C) Bar charts show mean ± SEM luciferase activity for each enhancer relative to the negative control (NegCTL) in Fujioka (upper panel) and THP1 (lower panel) AML cells (n = 3 biological replicates). (D) PCR amplification of each enhancer region shows deletion of the targeted enhancer. Red boxes indicate the PCR product expected from each deleted enhancer. (E) Mean ± SEM relative expression of the indicated genes, as determined by qPCR, following the indicated control or enhancer deletions (n = 3–5 biological replicates). ∗p < 0.05, ∗∗p < 0.01 using one-way ANOVA with Tukey post hoc test for the indicated comparisons. NS = not significant. See also Figure S2 and Table S1.

Making use of our prior ChIPseq datasets for H3K27Ac, H3K4Me1, and Mediator 1 from Fujioka AML cells,^15^ closer inspection revealed that the candidate regulatory region could be divided into four sub-regions (E1-4) based on the presence of separate Mediator 1 peaks. Region E3 exhibited three separate peaks and was subdivided into sub-regions E3A-C (Figures 2A and 2B). To determine the ability of these sequences to drive gene expression, we performed luciferase assays in Fujioka and THP1 AML cells, both of which express *IRX3* at high levels. Candidate enhancer sequences were inserted upstream of a minimal promoter controlling luciferase reporter gene expression. Both cell lines gave similar results. Region E1 exhibited the strongest activity, comparable to the positive control (an active SV40 enhancer sequence), with regions E2, E3A, E3C, and E4 all exhibiting activity above background. Region E3B gave results indistinguishable from the negative control (Figure 2C).

Next, we tested the functional relevance of the putative enhancer modules for the activation of *IRX3* expression in whole cells using CRISPR-Cas9 knockout. Fujioka cells were electroporated with CRISPR guide RNAs targeting each putative enhancer sequence in turn, a scrambled guide non-targeting RNA control (NTC) and, as a further control, CRISPR guide RNAs targeting a locus ∼80kB upstream of E1-E4 which was not acetylated, methylated, nor occupied by Mediator (Figure 2A). Efficient deletion of each enhancer was confirmed by PCR amplification of the targeted region (Figures 2D and S2A). Consistent with the luciferase assay results, and by comparison with the control CRISPR guides targeting an alternate site within the TAD, deletion of Region E1 led to a significant reduction in *IRX3* expression (to ∼25% of control) (Figure 2E). There was no effect on the expression of any other gene within the TAD. Deletion of E2 also led to a significant reduction in *IRX3* expression, although to a lesser extent (∼65% of control). Deletion of either E3 or E4 had no effect on gene expression. Thus, two regulatory elements within the final intron of *FTO* positively regulate the expression of *IRX3* in AML cells.

### Long non-coding transcripts arising from intron 8 of *FTO*

**Figure 3:**
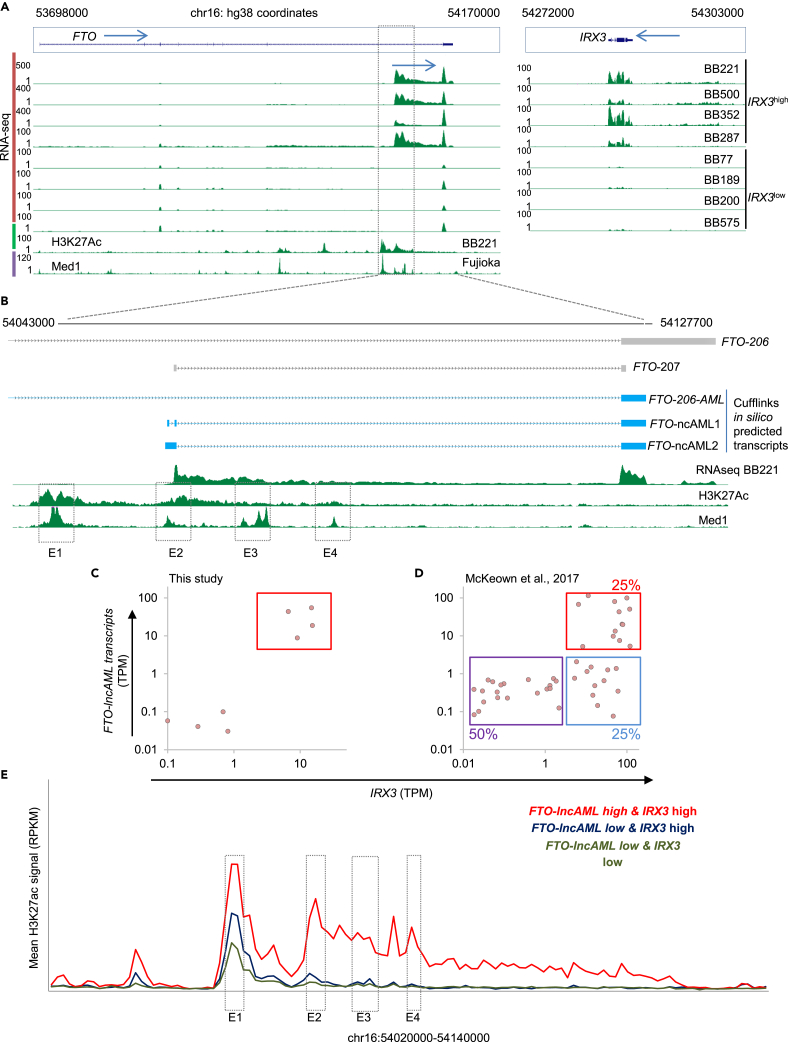
Unannotated transcripts arising from intron 8 of *FTO* in AML (A) RNAseq and ChIPseq tracks from the indicated cell line and primary patient samples. The candidate regulatory region is represented by dashed lines. Blue arrows indicate direction of transcription. (B) Diagram shows exon structure of the indicated annotated *FTO* transcripts (gray) and transcripts predicted by Cufflinks analysis (blue) and the RNAseq track from primary AML sample BB221 (green). (C and D) Scatterplots show the expression of *FTO*-lncAML transcripts versus *IRX3* in (C) our NPM1-mutated Biobank primary samples (n = 8) and (D) samples from McKeown et al.^14^ (n = 54). (E) Graph shows mean H3K27ac ChIP signal over the intron 8 candidate regulatory region for 13 *FTO*-lncAML^high^*IRX3*^*high*^ (red), 13 *FTO*-lncAML^low^*IRX3*^high^ (blue), and 28 *FTO*-lncAML^low^*IRX3*^low^ primary samples. Data are derived from McKeown et al.^14^ Areas corresponding to the enhancer modules are represented by dashed boxes. See also Figures S3, S4, and Table S1.

To better elucidate the mechanisms by which FTO intron 8 elements regulate *IRX3* activation, we analyzed RNAseq data from the primary AML samples used for our 4Cseq and ChIPseq experiments and noted transcripts arising from putative enhancer E2 running through to the last exon of *FTO*. These were only detected in *IRX3*^high^ samples (Figure 3A). To further characterize *FTO* locus transcripts, in the first instance we analyzed STAR-aligned reads with Cufflinks^16^^,^^17^ to deduce transcripts *in silico* through *ab initio* transcriptome assembly. After removing single exon sequences, the analysis suggested the presence of three transcripts that overlapped with but did not precisely map to previously annotated transcripts *FTO*-206 and *FTO*-207 (Figure 3B). *FTO*-206 is the MANE transcript (for Matched Annotation from NCBI and EMBL-EBI transcript) for *FTO*, and *FTO*-207 is a two exon non-coding transcript (ENST00000472835) hitherto identified from only a single expressed sequence tag.^18^ Cufflinks analysis suggested that all three *FTO* transcripts in AML cells overlapped partially with exon 9 and that two, arising from close to putative enhancer E2, overlapped partially with *FTO*-207. We termed these *in silico* predicted transcripts *FTO*-206-AML, *FTO*-lncAML1. and *FTO*-lncAML2. The non-coding transcripts arising from intron 8 of *FTO* were predicted by Cufflinks to contain three and two exons, respectively (Figure 3B), in keeping with their being long non-coding transcripts.^19^ We confirmed the presence of *FTO*-lncAML transcripts in RNA sequencing analyses from an additional dataset,^14^ and their association with high *IRX3* expression (Figures 3C, 3D, and S3A) using Kallisto analysis^20^: all AML cases with high *FTO*-lncAML expression exhibited high *IRX3* expression, whereas in the reverse analysis 50% of *IRX3*^high^ AML samples expressed *FTO*-lncAML-transcripts.

The observation of *FTO*-lncAML^low^ *IRX3*^high^ AML cases prompted us to evaluate how H3K27Ac across the *IRX3* TAD was associated with the presence or absence of *FTO*-lncAML transcripts. *IRX3*^high^ AML cases exhibited significantly higher H3K27Ac by comparison with *IRX3*^low^ cases at sites surrounding *IRX3* and *IRX5* whether or not *FTO*-lncAML was expressed (Figure S3B). However, increased acetylation within intron 8 of *FTO*, and acetylation at additional sites in introns 6 and 7, was only seen where *FTO*-lncAML was expressed (Figures 3E and S3B), and this was not explained by differences in mutation status (Table S1). Interestingly, evaluation of H3K27Ac patterns in normal CD34^+^ HSPCs—where *FTO*-lncAML is not expressed (Figure S4A)—revealed a pattern qualitatively similar to that seen in *FTO*-lncAML^low^ *IRX3*^low^ AML cases (Figures S3B and S4B).^21^ Furthermore, the H3K27Ac peak corresponding to location E1 seen in normal CD34^+^ HSPCs was lost in mature downstream hematopoietic cells (Figure S4B).^22^^,^^23^ Thus, in human AML cases with high *IRX3* expression there are two patterns of histone acetylation within the *IRX3* TAD: in *FTO*-lncAML^low^ cases increased H3K27Ac is found surrounding *IRX3*, *CRNDE*, and *IRX5* whereas in *FTO*-lncAML^high^ cases there is in addition significant H3K27Ac ChIP signal within distal intronic sequences of *FTO*.

### Sequence determination of *FTO*-lncAML transcripts

**Figure 4:**
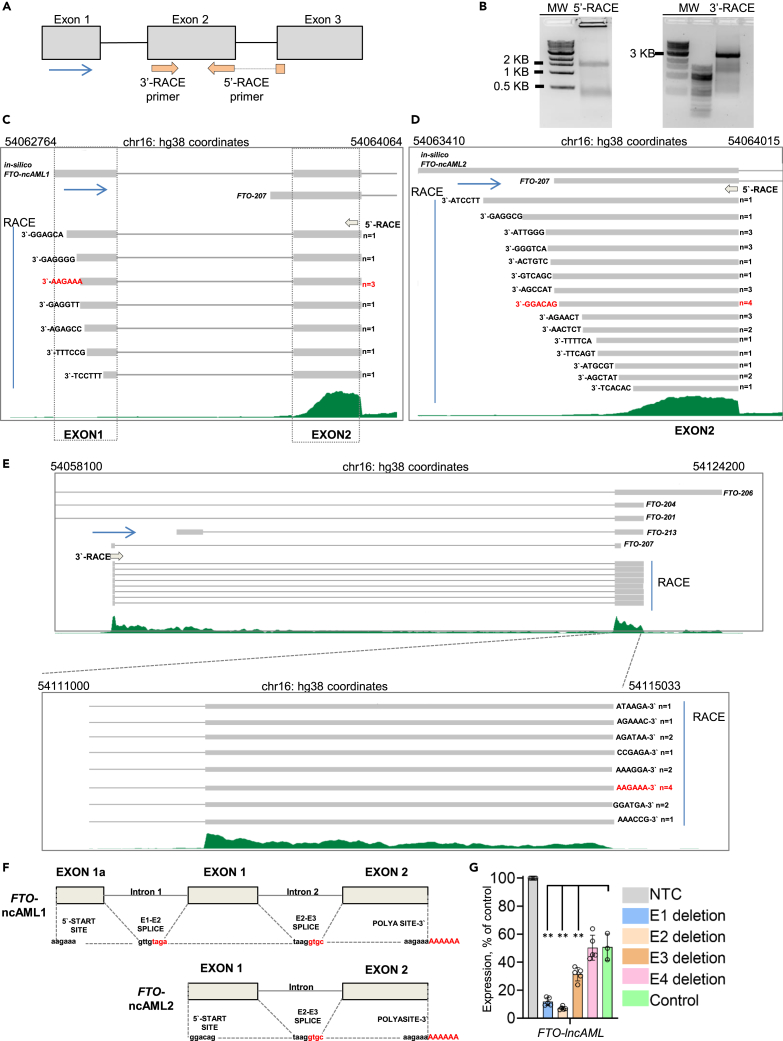
Sequence determination of *FTO*-lncAML transcripts (A) Schematic representation of Cufflinks-predicted exon structure of *FTO*-lncAML1 showing primers used for RACE. Blue arrow indicates direction of transcription. (B) Amplification products obtained from 5’ (left) and 3’ (right) RACE. (C and D) Schematic representation of the sequencing results of the 5′-cDNA amplification showing transcripts predicted to have at least (C) three or (D) two exons. Sequences are shown above the RNAseq track from primary AML sample BB221, along with exon structure of the *in silico* predicted transcripts and *FTO*-207. The most commonly identified sequenced clones are in red. Blue arrows indicate direction of transcription. (E) Schematic representation of sequencing results for the 3′-cDNA amplification. Upper panel: exon structure of identified RACE-transcripts and previously annotated *FTO* transcripts. Lower panel: sequence of 3′ clones, with the most commonly seen 3′ sequence in red. (F) Schematic representation of exon and intron structure of the commonest FTO-lncAML isoforms. The 5′-start site, 3′-termination site and splice sites are indicated. (G) Mean ± SEM relative expression of *FTO*-lncAML as determined by qPCR after CRISPR deletion of the indicated regulatory elements in Fujioka AML cells (n = 3–5 biological replicates). ∗p < 0.05, ∗∗p < 0.01, using one-way ANOVA with Tukey post hoc test. See also Figure S5.

Next, to determine the actual rather than predicted full-length sequence of *FTO*-lncAML transcripts, we performed rapid amplification of cDNA ends (RACE) in Fujioka AML cells (Figures 4A and 4B), making use of the Cufflinks-predicted exon structure of *FTO*-lncAML to design appropriate primers. The 5′-RACE revealed two transcripts. The first (represented by 9/37 sequenced clones) corresponded well with the predicted *FTO*-lncAML1, exhibiting a three-exon structure (Figure 4C). The second more prevalent two-exon transcript (represented by 28/37 of sequenced clones) however exhibited a start site that aligned well with the previously annotated *FTO*-207 transcript rather than the Cufflinks-predicted transcript (Figure 4D). The 3′-RACE confirmed similar transcript termination sites in 14/28 sequenced clones (Figure 4E) which matched the termination sites of previously annotated transcripts *FTO*-201 (ENST00000268349), *FTO*-204 (ENST00000463855) and *FTO*-213 (ENST00000636091), but not *FTO*-206 or *FTO*-207. The remaining 14 sequenced clones exhibited more variable distal sequences with a range of splicing patterns (Figure S5A). All transcripts were polyadenylated. Based on these analyses we concluded that the two-exon *FTO*-lncAML2 was most likely to be the most frequent species of transcript (Figure 4F).

Transcripts were assessed as having very low protein-coding potential using the Coding Potential Calculator 2 (CPC2) as was control non-coding RNA *XIST*, while the protein-coding isoform *FTO-*206 and Glyceraldehyde-3-Phosphate Dehydrogenase (GAPDH) mRNA were readily identified as protein-coding RNA species (Figure S5B). Likewise, while the protein-coding transcript for *GAPDH* was predominantly located in the cytoplasm in sub-cellular fractionation assays, both U6 small nuclear RNA and *FTO*-lncAML transcripts were predominantly located in the nucleus (Figure S5C).

To determine whether the expression of *FTO*-lncAML was controlled by the putative enhancer modules E1-E4 (Figure 2B), we performed additional quantitative PCR on our CRISPR deleted samples (Figure 2E). By comparison with the control CRISPR sample making use of guides targeting an alternate site within the TAD, *FTO*-lncAML expression (determined using primers bridging exons 1 and 2; Figure 4E) was substantially reduced (to ∼15% of control) by deletion of E2, which was as expected because this results in the destruction of its transcription start site (Figure 4G). Moreover, deletion of E1 and E3 also significantly reduced *FTO*-lncAML expression (respectively to ∼23% and 60% of control values) (Figure 4G).

Thus, in AML cells bioinformatics analysis and RACE experiments indicate the presence of multiple isoforms of polyadenylated transcript arising from close to putative enhancer E2. These isoforms the most frequent of which we term *FTO*-lncAML1 and 2 likely predominantly include three or two exons, respectively (Figure 4F).

### *FTO*-lncAML positively regulates *IRX3* expression in AML cells

**Figure 5:**
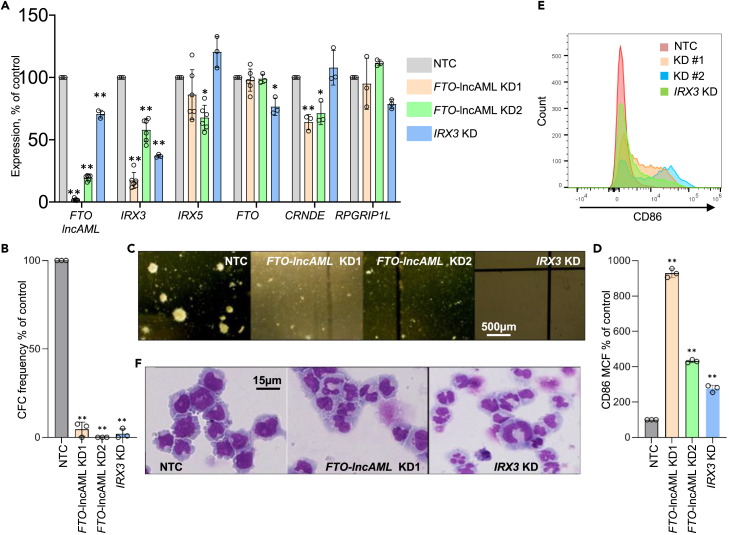
*FTO*-lncAML regulates *IRX3* expression and retards differentiation in AML cells (A) Mean ± SEM relative expression of the indicated genes as determined by qPCR 96 h following initiation of KD of *FTO*-lncAML or *IRX3* in Fujioka AML cells. (B) Bar chart shows mean ± SEM colony forming cell (CFC) frequency relative to control cells in the indicated conditions. (C) Representative images of colonies. (D) Bar chart shows mean ± SEM CD86 mean cell fluorescence (MCF) as determined by flow cytometry on day 6 in the indicated conditions. (E) Representative flow cytometry plots. (F) Representative images of cytospins of cells from Day 7. For (A), (B), and (D) ∗p < 0.05, ∗∗p < 0.01, using one-way ANOVA with Fisher’s least significant difference post hoc test (n = 3–6 biological replicates). See also Figure S6.

Next, to evaluate the functional effect of *FTO*-lncAML in controlling the expression of genes within the *IRX3* TAD, we performed shRNA-mediated KD in Fujioka AML cells of *FTO*-lncAML using two shRNAs (KD1 and KD2) targeting exon 1 (Figure 4F), thereby avoiding targeting exon 2 which is shared with some full-length *FTO* transcripts. Both KD1 and KD2 substantially downregulated expression of *FTO*-lncAML, although with differing efficiencies (Figure 5A). KD of *FTO*-lncAML led to proportionate downregulation of *IRX3* and to a lesser extent *CRNDE* (Figure 5A). KD of *IRX3* to ∼35% of control levels modestly reduced expression of both *FTO* and *FTO*-lncAML, but not that of any other genes within the *IRX3* TAD (Figure 5A). Both *FTO*-lncAML and *IRX3* KD led to markedly reduced clonogenic activity of Fujioka AML cells in semi-solid culture (Figures 5B and 5C), with upregulation of the monocyte/macrophage differentiation marker CD86 (Figures 5D and 5E), morphological features of differentiation in cytospin analyses (Figure 5F) and modest apoptosis in the case of the *FTO*-lncAML KD1 construct (Figure S6A).

**Figure 6:**
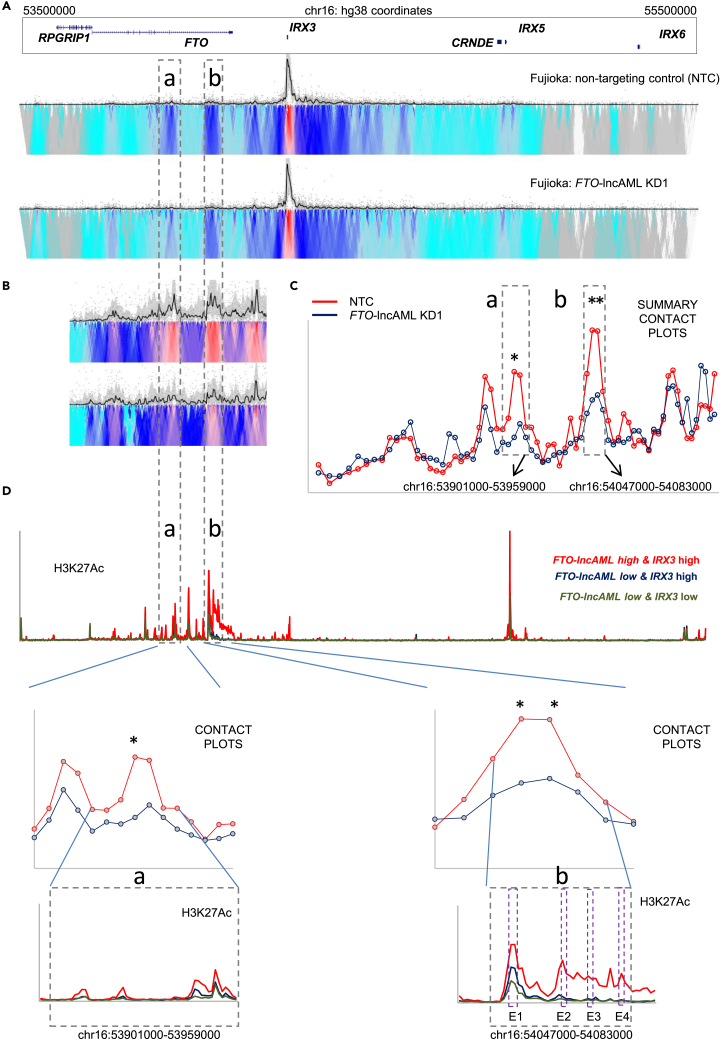
*FTO*-lncAML promotes contact between intron 8 of FTO and the *IRX3* promoter (A) Representative 4Cseq plots of Fujioka cells 96 h following initiation of KD of *FTO*-lncAML. The top panel shows data from a non-targeting control (NTC) while the bottom shows *FTO*-lncAML KD. Regions with differential contact strength are defined as a and b. (B) Regions with differential 4Cseq contact strength are highlighted. (C) Plot shows mean normalized 4Cseq signal counts from two biological replicates of control (NTC) and *FTO*-lncAML KD1 Fujioka cells. ∗ indicates p < 0.01 by t-test for the indicated loci. (D) Enhanced view aligning histone acetylation with summary contact plots.

We evaluated localized changes in chromatin conformation and epigenome modifications upon *FTO*-lncAML KD in Fujioka AML cells. Prior studies have indicated that one role of enhancer origin RNAs is to promote looping between enhancers and promoters.^24^^,^^25^ We performed 4Cseq using the *IRX3* promoter as a bait with and without *FTO*-lncAML KD and noted a strong reduction of the interaction between the *FTO* intron 8 regulatory region and the *IRX3* promoter (Figures 6A–6D). The reduced contacts following *FTO*-lncAML KD were also associated in ChIP-PCR analyses with reduced H3K27Ac ChIP signal at both the *FTO* intron 8 regulatory region and the *IRX3* promoter (Figure S6C).

### Association of *FTO*-lncAML expression with expression of a stem cell program

**Figure 7:**
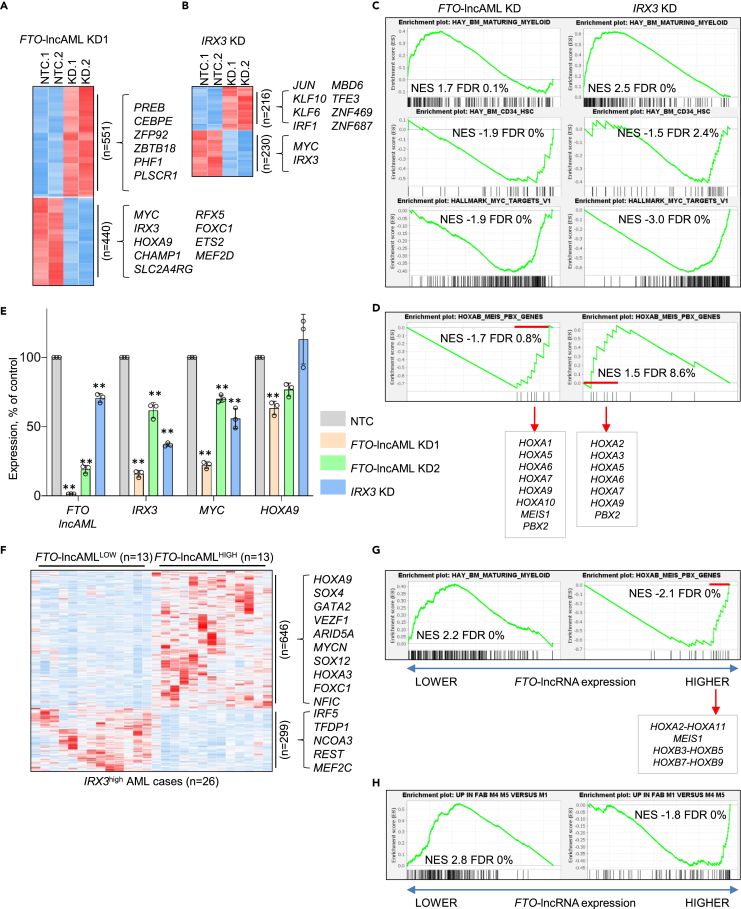
Association of *FTO*-lncAML with HOXA gene expression (A and B) Heatmaps represent differentially expressed genes in Fujioka AML cells 96 h following initiation of (A) *FTO*-lncAML or (B) *IRX3* KD. Differentially expressed transcription factor genes are highlighted. (C and D) GSEA plots show enrichment of the indicated gene sets in the indicated conditions. NES, normalized enrichment score; FDR, false discovery rate. In (D) leading edge genes are highlighted. (E) Mean ± SEM relative expression of the indicated genes as determined by qPCR 96 h following initiation of KD of *FTO*-lncAML or *IRX3* in Fujioka AML cells. ∗p < 0.05, ∗∗p < 0.01, using one-way ANOVA with Fisher’s least significant difference post hoc test (n = 3 biological replicates). Note that results for *FTO*-lncAML and *IRX3* are reproduced here from Figure 5A to facilitate interpretation of data on *MYC* and *HOXA9*. (F) Heatmap represents differentially expressed genes in *FTO-lncAML*^high^*IRX3*^high^ (n = 13) and *FTO*-lncAML^low^*IRX3*^high^ (n = 13) primary AML cases. Data are derived from McKeown et al.^14^ Differentially expressed transcription factor genes are highlighted. (G and H) GSEA plots show enrichment of the indicated gene sets in *FTO*-lncAML^low^*IRX3*^high^ versus *FTO*-lncAML^high^*IRX3*^high^ patient samples. the indicated conditions. (G) In the right hand panel leading edge genes are highlighted. See also Tables S2 and S3.

To further evaluate the consequences of *FTO*-lncAML or *IRX3* transcript depletion on the transcriptome of Fujioka AML cells, we performed RNA sequencing of cells following KD. Considering expressed protein-coding genes (i.e., those with expression levels of 0.5 fragments per kilobase per million mapped reads [FPKM] in at least one of the samples; n = 7999 for *FTO*-lncAML KD1, n = 8141 for IRX3 KD), 991 or 446 were differentially expressed 72 h after initiation of *FTO*-lncAML or *IRX3* KD, respectively (Figures 7A and 7B): 551 or 216 were upregulated and 440 or 230 downregulated (Figures 7A, 7B, and Table S2). Comparison of the changes in gene expression induced by the two separate KDs revealed similarities as well as some significant differences. As expected, and in keeping with our colony forming and differentiation assay data (Figure 5), Gene Set Enrichment Analysis (GSEA) confirmed that in both cases there was upregulation of a maturing myeloid differentiation program, and downregulation of genes expressed in CD34^+^ hematopoietic stem and progenitor cells, and genes which are targets of MYC (Figure 7C). However, we noted that *HOXA9* was among the downregulated genes following *FTO*-lncAML KD but not *IRX3* KD (Figures 7A and 7B). Using HOXA, HOXB, MEIS, and PBX genes as a gene set, in additional GSEA we noted that while *FTO*-ncRNA KD led to coordinate downregulation of HOXA genes in particular, the opposite was the case following *IRX3* KD (Figure 7D). In separate experiments, we confirmed by qPCR that *FTO*-lncAML KD but not *IRX3* KD resulted in downregulation of *HOXA9*, while *MYC* transcripts were downregulated in both cases (Figure 7E).

To determine whether expression of *FTO*-lncAML in primary AML cells was associated with differential expression of HOX genes, we divided *IRX3*^high^ AML samples^14^ into those with high or low *FTO*-lncAML expression and noted that there were significant differences in gene expression not explained by mutation profile (Table S1) (Figure 7F). Among genes more highly expressed in *FTO*-lncAML^high^ IRX3^high^ AML samples (n = 646; Table S3) in particular, there were significant enrichments for genes with a “Transcription” Biological Process Gene Ontology annotation (p = 10^−3^). Among genes more highly expressed in *FTO*-lncAML^low^ IRX3^high^ AML samples (n = 299) there were significant enrichments for genes with the Cellular Component Gene Ontology annotation “Plasma membrane” (p = 10^−5^). Notably, there was significantly higher expression of transcription factor genes *HOXA9*, *GATA2*, and *SOX4* (Figure 7F). In keeping with the Gene Ontology assessment, GSEA revealed that *FTO*-lncAML^high^ *IRX3*^high^ AML samples expressed lower levels of genes upregulated in maturing myeloid cells by comparison with *FTO*-lncAML ^low^ IRX3^high^ AML samples and higher levels of *HOXA* and *HOXB* genes (Figure 7G). Likewise, *FTO*-lncAML^high^ *IRX3*^high^ AML samples expressed lower levels of genes highly expressed in AML of myelomonocytic or monocytic/monoblastic (M4 or M5) subtypes by comparison with AML with minimal differentiation (M1), and vice versa for *FTO*-lncAML ^low^ IRX3^high^ AML samples (Figure 7H).

Thus, in patient samples, expression of *FTO*-lncAML associates with a less well-differentiated transcriptional state featuring a higher expression of stem cell genes, and following *FTO*-lncAML but not IRX3 KD, HOXA genes are downregulated in cell line models.

## Discussion

Our genome editing, epigenomic profiling, and long-range chromatin interaction analyses reveal that sequences within intron 8 of *FTO* positively regulate *IRX3* in AML. These are distinct from the polymorphic intronic sequences of *FTO* associated with human obesity which have been found to regulate *IRX3* expression in adipocytes and hypothalamic neurons. High-level expression of *IRX3* is frequent in human leukemia, is sufficient for immortalization of murine hematopoietic stem and progenitor cells, and when co-expressed with *Hoxa9* in a murine model substantially deepens the level of differentiation block observed in the resulting AMLs.^11^ Our analysis of histone acetylation patterns in *IRX3*^high^ primary AML samples shows that around half of cases exhibit strong H3K27ac ChIP signal across a ∼32kB region within intron 8 of *FTO*. By comparison with *IRX3*^high^ primary AML cases which lack strong intron 8 *FTO* acetylation, intron 8 acetylated cases are less well differentiated based on their gene expression profile, and express higher levels of stem cell genes such as *HOXA9*, *GATA2*, and *SOX4*, even while they do not differ significantly from a mutational viewpoint. The reasons why AMLs with similar mutational profiles exhibit different levels of differentiation block are likely to be heterogeneous. Different patterns of expression of key transcriptional regulators might perhaps be related to the occurrence of initiating mutations in different cells of origin, genetic polymorphisms, or alternate patterns of DNA methylation. Indeed, the intron 8 regulatory region in *IRX3*^high^ primary AML cases with strong intron 8 H3K27ac is DNA hypomethylated, although whether hypomethylation is permissive for or merely accompanies enhancer activation remains unclear.

Each of the four distinct modules within the regulatory region contains sequences that readout as putative enhancers in luciferase assays to variable extent, E1 being the most potent. However, following CRISPR knockouts only E1 and E2 deletion served to reduce *IRX3* expression significantly, perhaps consistent with a hierarchical model of enhancer cluster:gene activation as has been described elsewhere.^26^ According to this pattern, one module within a cluster drives a much larger effect on the target gene than the others, although in this case E1 would appear to exhibit more traditional enhancer activity toward both *IRX3* and *FTO*-lncAML whereas E2 may potentially serve to enhance *IRX3* expression entirely through serving as the transcriptional start site for *FTO*-lncAML. More generally, other questions about the architecture and function of this region remain unanswered, and this highlights the challenge associated with the functional characterization of clustered regulatory elements. For example, it remains to be determined whether the modules contribute to the activation of *IRX3* synergistically or additively.

*IRX3*^high^ primary AML cases with strong histone acetylation of intron 8 of FTO also exhibit expression of long non-coding, spliced, and polyadenylated transcripts around 3kB in length arising from the same locus; as expected, deletion of E1-E3 in an AML cell line substantially reduced their expression. While *FTO*-lncAML is transcribed from an enhancer region it is not a typical enhancer RNA because it is spliced, >1kB in length, and polyadenylated—features which are consistent with it being an enhancer lncRNA or e-lncRNA.^19^ Intriguingly, it appears integrally involved in the ability of this regulatory region to control the expression of *IRX3* because its KD led to reduced contacts between intron 8 of *FTO* and the promoter of *IRX3*, reduced intron 8 H3K27ac and reduced expression of *IRX3*, while largely sparing the expression of other genes within the TAD. The mechanism currently remains unclear, although there is an increasing appreciation of the importance of RNA in regulating the activity and interactions of transcription factors.^27^^,^^28^^,^^29^ Furthermore *cis*-acting and activating long non-coding RNAs have been reported to modulate enhancer activity through recruiting CCCTC-binding factor (CTCF), altering the nuclear position of an enhancer and recruitment of enhancer activating proteins.^19^ Our observation is similar to that of Blinka and colleagues who found that a regulatory cluster 45kB upstream of *Nanog* activates expression of both *Nanog* and the neighboring gene *Dppa3*, but KD of an enhancer-associated transcript only affected the expression of *Dppa3*.^30^

While both KD of *FTO*-lncRNA and *IRX3* led to AML cell differentiation and downregulation of *MYC*, there was discordance in the consequences of these knockdowns for HOXA gene expression. Related to this, *FTO*-ncRNA expressing cases of IRX3^high^ AML expressed higher levels of HOX genes, other stem cell genes such as *GATA2* and *SOX4*, and *FOXC1* which we have previously shown also contributes to impaired differentiation in AML.^31^ Thus, *FTO*-lncRNA may have activity *in trans*, through mechanisms unknown, over and above its role in sustaining *IRX3* expression. Such a mechanism has previously been postulated for the activity of the long non-coding RNA *LncHSC-2* in hematopoietic stem cells.^32^ Recently, Arza-Apalategi and colleagues^33^ also noted the association of *FTO*-lncRNA expression (which they termed IFEX9) with the presence of a higher level of differentiation block in NPM1-mutated cases within the Beat AML cohort, although this was not linked functionally to either *IRX3* or *HOXA* gene expression.

*IRX3* is also strongly expressed in up to 50% of cases of human T-acute lymphoblastic leukemia, and its forced expression in normal T cell precursors impedes their differentiation *in vitro*.^11^ Interestingly, in a subset of these cases heterozygous deletions of a region within intron 8 of *FTO* have been identified in both primary T-ALL cases and in cell lines. These deletions encompass a CTCF binding site and experimental disruption of this site led to increased *IRX3* expression in *IRX3*^negative^ PF-382 T-ALL cells though an enhancer hijacking mechanism, with expanded contacts with the *CRNDE*-*IRX5* locus which is strongly acetylated during normal T cell development.^34^ These findings may provide insight into our observation that around 50% of *IRX3*^high^ cases of AML lack expression of *FTO*-lncAML; here *IRX3* expression might be promoted through interaction with the *CRNDE*-*IRX5* locus rather than intron 8 of *FTO*, although once more the upstream mechanisms driving activation are unclear.

In summary, we report the identification and characterization of a regulatory cluster and its associated transcripts active in ∼10% of cases of human AML which sits within intron 8 of *FTO* and which activates *IRX3* and HOX genes.

### Limitations of the study

One limitation is that our conclusions regarding the phenotypic and molecular consequences of *FTO*-lncAML depletion arise from experiments performed in an AML cell line setting; helpful additional information might be gleaned from technically more challenging experiments in primary human AML cells using both *in vitro* and *in vivo* approaches. Moreover, even though we observed reduced interaction between intron 8 of FTO and the promoter of IRX3, and reduced acetylation of both enhancer modules and the IRX3 promoter upon depletion of *FTO*-lncAML, the detailed mechanism of action, including any potential *in trans* activity, of the lncRNA is currently unknown and requires further investigation. Finally, the mechanism that we propose for the activation of *IRX3* in AML cannot explain how this transcription factor gene is so highly expressed in around 50% of cases of T-acute lymphoblastic leukemia, where expression of *FTO*-lncAML is not observed.

## STAR★Methods

### Key resources table

**Table:** 

REAGENT or RESOURCE	SOURCE	IDENTIFIER
**Antibodies**		

anti-CD86-PerCP-eFlour710	ThermoFisher	Cat#46086282; RRID:AB_2815140
anti-acetyl H3K27	Abcam	Cat#ab4729; RRID:AB_2118291
anti-monomethyl-H3K4	Abcam	Cat#ab8895; RRID:AB_306847
anti-MED1	Bethyl	Cat#A300-793A RRID: AB_577241
5-methylcytosine monoclonal antibody 33D3	DIAGENODE	Cat#C15200081-100 RRID: AB_2572207

**Bacterial and virus strains**		

One shot Stbl3 Chemically Competent E.col	ThermoFisher	Cat#C737303
Stellar™ Competent Cells	TAKARA BIO	Cat#636766

**Biological samples**		

Primary human AML samples	Manchester Cancer Research Centre Tissue Biobank	the-christie.biobank@nhs.net

**Chemicals, peptides, and recombinant proteins**		

Puromycin	Sigma	Cat#P8833
Methylcellulose medium	Stem Cell Technologies	Cat#04230
Dynabeads Protein G	Invitrogen	Cat#10004D
T4 DNA Ligase	NEB	Cat#M0202L
T4 DNA Ligase	Roche	Cat#799009
Polybrene	Milipore	Cat#TR1003G
Trypan Blue Solution	GIBCO	Cat#15250-061
ChIP Crosslink Gold	Diagenode	Cat#C01019027
SYBR Green	ThermoFisher	Cat#4309155
May-Grunwald Giemsa	Sigma	Cat#MG500
Alt-R Cas9	Integrated DNA Technologies	Cat#1081059
XbaI	NEB	Cat#R0145L
SbfI	NEB	Cat#R3642L
Age1	NEB	Cat#R3552S
EcoR1-HF	NEB	Cat#R3101L
DpnII	NEB	Cat#R0543S
NlaIII	NEB	Cat#R0125S

**Critical commercial assays**		

RNeasy Plus Micro Kit	QIAGEN	Cat#74034
RNeasy Plus Mini Kit	QIAGEN	Cat#74134
High-Capacity cDNA Reverse Transcription	ThermoFisher	Cat#4368814
APC Annexin Kit	BD Pharmigen	Cat#550474
DNeasy blood and tissue kit	QIAGEN	Cat#69504
QIAamp DNA Blood Mini Kit	QIAGEN	Cat#51104
QIAshredder	QIAGEN	Cat#79656
QIAquick PCR Purification Kit	QIAGEN	Cat#28104
Neon Transfection System	ThermoFisher	Cat# MPK1096
Dual-Glo Luciferase Assay System	Promega	Cat#E2920
Microplex Library Preparation Kit	Diagenode	Cat#C05010012
NEB Next Ultra II DNA Library Prep Kit for Illumina	NEB	Cat#E7645S
MagMeDIP kit	DIAGENODE	Cat#C02010021
IPure kit v2	DIAGENODE	Cat#C03010015
SMARTer® RACE 5’/3’ Kit	TAKARA BIO	Cat#634858
In-Fusion® HD Cloning Kit	TAKARA BIO	Cat#639650
TURBO DNA-free™ Kit	ThermoFisher	Cat#AM1907
TruSeq Stranded Total RNA with Ribo-Zero Globin	Illumina	Cat#20020612
SureSelect PolyA	Agilent	Cat# 5190-6411
Kapa Library Quantification Kit for Illumina sequencing	Roche	Cat#07960140001

**Deposited data**		

H3K27Ac ChIP-seq from McKeown dataset	McKeown et al.,^14^	SRP103200
RNA-seq from McKeown dataset	McKeown et al.,^14^	SRP103200
TS49_ H3K27Ac_ NTC	Simeoni et al.,^15^	GSM4837697
TS83_H3K4me1	Simeoni et al.,^15^	GSM4837705
DNA and RNA sequencing data produced in this study	This study	GEO: GSE223792
TS116_05_287_H3K27Ac	This study	GSM7049777
TS116_06_352_H3K27Ac	This study	GSM7049778
TS116_04_221_H3K27Ac	This study	GSM7049776
TS116_07_500_H3K27Ac	This study	GSM7049779
TS116_02_189_H3K27Ac	This study	GSM7049774
TS116_03_200_H3K27Ac	This study	GSM7049775
TS116_01_77_H3K27Ac	This study	GSM7049773
TS116_09_INPUT	This study	GSM7049781
TS108_1_MeDIP_189	This study	GSM7049782
TS108_2_MeDIP_200	This study	GSM7049783
TS108_3_MeDIP_352	This study	GSM7049784
TS108_4_MeDIP_500	This study	GSM7049787
TS108_5_INPUT_200	This study	GSM7049785
TS108_6_INPUT_500	This study	GSM7049786
TS112_1_RNA_77	This study	GSM7075847
TS112_2_RNA_189	This study	GSM7075848
TS112_3_RNA_200	This study	GSM7075849
TS112_4_RNA_221	This study	GSM7075850
TS112_5_RNA_287	This study	GSM7075851
TS112_6_RNA_352	This study	GSM7075852
TS112_7_RNA_500	This study	GSM7075853
TS112_8_RNA_575	This study	GSM7075854
TS105_05_Fujioka-MED1	This study	GSM7421875
TS160_06_FTO_NTC-1	This study	GSM6995406
TS160_04_FTO_NTC-2	This study	GSM6995407
TS160_08_FTO_KD-1	This study	GSM6995408
TS160_02_FTO_KD-2	This study	GSM6995409
TS160_07_IRX3_NTC-1	This study	GSM6995410
TS160_01_IRX3_NTC-2	This study	GSM6995411
TS160_05_IRX3_KD-1	This study	GSM6995412
TS160_03_IRX3_KD-2	This study	GSM6995413
TS103_287T1M_A_IRX3_ high_VE_TAGCTT_ GAAGGTGGGCGCCCGATC	This study	GSM6995414
TS103_352T1B_C_IRX3_ high_VE_CTTGTA_ GAAGGTGGGCGCCCGATC	This study	GSM6995415
TS103_575T1M_F_IRX3_ low_VE_TGACCA_ GAAGGTGGGCGCCCGATC	This study	GSM6995416
TS103_189T2M_E_IRX3_ low_VE_TTAGGC_ GAAGGTGGGCGCCCGATC	This study	GSM6995417
TS161_Fujioka_NTC-1_VPE_TAGCTT_GCTC CGGACGTGCGGTAC	This study	GSM6995418
TS161_Fujioka_KD-1_VPE_GGCTAC_GCTCC GGACGTGCGGTAC	This study	GSM6995419
TS161_Fujioka_NTC-2_VPE_CTTGTA_GCTCC GGACGTGCGGTAC	This study	GSM6995420
TS161_Fujioka_KD-2_VPE_ACAGTG_GCTCCG GACGTGCGGTAC	This study	GSM6995421

**Experimental models: Cell lines**		

THP1	DSMZ	RRID:CVCL_0006
Fujioka	JCRB	RRID:CVCL_1632
HEK293	Invitrogen	RRID:CVCL_0045

**Oligonucleotides**		

See Table S4	This study	N/A

**Recombinant DNA**		

pLKO.1 - TRC cloning vector	Addgene	Cat#10878, RRID:Addgene_10878
pLKO.1 (SHC002)	Sigma aldrich	Cat#SHC002
pLKO.1: NTC	This study	N/A
pLKO.1: FTO-lncAML KD1	This study	N/A
pLKO.1: FTO-lncAML KD2	This study	N/A
pLKO.1: IRX3 KD	Somerville et al.,^11^	N/A
pLS-mP-Luc	Addgene	Cat#106253, RRID:Addgene_106253
pLS-mP-Luc: SV40 enhancer	This study	N/A
pLS-mP-Luc: ACTB genomic locus	This study	N/A
pLS-mP-Luc: E1	This study	N/A
pLS-mP-Luc: E2	This study	N/A
pLS-mP-Luc: E3-A	This study	N/A
pLS-mP-Luc: E3-B	This study	N/A
pLS-mP-Luc: E3-C	This study	N/A
pLS-mP-Luc: E4	This study	N/A
pLS-SV40-mP-Rluc	Addgene	Cat#106292, RRID:Addgene_106292

**Software and algorithms**		

STAR	Dobin et al.,^16^	N/A
BWA-MEM version 0.7.15	Li and Durbin,^35^	N/A
MEDIPS	Lienhard et al.,^36^	N/A
DESeq2	Love et al.,^37^	N/A
SDS software v2.1	Applied Biosystems	N/A
Samtools	Li et al.,^35^	N/A
Cutadapt	Martin, 2012	N/A
GSEA v2.0.14	Subramanian et al., 2005	N/A
Microsoft Excel 2007	N/A	N/A
Cufflinks v2.2.1	Trapnell et al.,^17^	N/A
Kallisto	Bray et al.^20^	N/A

### Resource availability

#### Lead contact

Further information and requests for resources and reagents should be directed to and will be fulfilled by the lead contact, Tim Somervaille (tim.somervaille@cruk.manchester.ac.uk).

#### Materials availability

• This study did not generate new unique reagents.
• All plasmids listed in the key resources table are available upon request.

### Experimental model and study participant details

#### Human tissue and cell lines

Use of human tissue was in compliance with the ethical and legal framework of the UK’s Human Tissue Act, 2004. Primary human AML samples were from Manchester Cancer Research Centre’s Tissue Biobank (instituted with the approval of the South Manchester Research Ethics Committee; 18/NW/0092). Their use was authorized following ethical review by the Tissue Biobank’s scientific sub-committee and with the informed consent of the donor. THP1 cells were purchased from DMSZ (Braunschweig, Germany). Fujioka cells were a gift from Dr. Vaskar Saha (Children’s Cancer Group, Manchester Cancer Research Centre). Both cell lines were verified by STR analysis and confirmed to be mycoplasma-free.

#### Primary AML cells and cell line cultures

THP1 (male) and Fujioka (male) cells were cultured as per DSMZ recommendations in Roswell Park Memorial Institute (RPMI) 1640 medium (Sigma Aldrich, St Louis, MO) supplemented with 2mM L-Glutamine (Life Technologies, Warrington, UK), 10% fetal bovine serum (FBS) (Sigma Aldrich) and 1% penicillin/streptomycin (Sigma Aldrich). HEK293 cells were cultured in Dulbecco's Modified Eagle's Medium (DMEM) (Sigma Aldrich, St Louis, MO) with the addition of 2mM L-Glutamine (Life Technologies, Warrington, UK) and 10% fetal bovine serum (FBS) (Sigma Aldrich). For semisolid culture, cells were grown in methylcellulose medium (H4320, Stem Cell Technologies, Vancouver, Canada) at a starting density of (2.5x10^3^/ml). Puromycin (Sigma Aldrich) was used at 3μg/ml and colonies were enumerated after 7-10 days. Cell lines were mycoplasma-free and authenticated by short tandem repeat DNA profiling.

Cryopreserved primary AML cells were thawed in a 37°C water bath and centrifuged at 200xg for 10 minutes in Minimum Essential Medium with alpha modification (α-MEM) supplemented with 12.5% heat-inactivated FBS (Life Technologies) and 12.5% heat-inactivated horse serum (Life Technologies) prior to use in high sequencing experiments.

### Method details

#### Viral particle manufacture for shRNA knockdowns and luciferase assay

To generate lentiviral particles, HEK293 cells were plated at 4x10^6^ cells/ plate in a 10cm dish and incubated overnight. Next day transfection was performed with 21μg of polyethylenimine 25kD (PEI), 4μg of lentiviral construct (pLKO.1 for shRNA KD or luciferase plasmids), 2μg of pCMVD8.91 and 1μg of pMDG.2. PEI and the plasmid solution were mixed and incubated at room temperature for 30 minutes before being added dropwise to the HEK293 plate and incubated overnight. Next day, medium was changed and cells incubated for 24 hours. Subsequently, HEK293 supernatant containing lentiviral particles was harvested for two consecutive days. For each harvest the supernatant was filtered through a 0.45μm filter (Nalgene). At this point, lentivirus supernatant was used for cell infections or stored at -80°C. For cell infections, 1.5x10^6^ cells were re-suspended in 100μl of R10 medium and mixed with 6ml of lentiviral supernatants supplemented with Polybrene (8μg/ml). Cells were centrifuged at 900xg for 30 minutes at 37°C and then incubated overnight. For shRNA KD, the following day cells were supplemented with 4ml of R10 medium and selected with puromycin at a final concentration of 3μg per ml. After 48 hours selected samples were used for the specific downstream applications.

#### shRNA design and cloning for lentiviral transduction

To generate lentiviral knockdown constructs targeting *FTO*-lncAML, single 21-mer oligonucleotides were selected using the Whitehead Institute's design site (http://sirna.wi.mit.edu/home.php).

The sense and antisense sequence for each 21-mer were used to generate oligos for cloning into pLKO.1 as follows:

FORWARD 5’ ccgg—21mer sense—ctcgag—21mer antisense—tttttg 3’

REVERSE 5’ aattcaaaaa—21mer sense—ctcgag—21mer antisense 3’

Oligonucleotide sequences are shown in Table S4. Oligos were annealed by incubating at 98°C for 5 mins with slow cooling to room temperature and then ligated into pLKO.1 puro (Addgene plasmid #10878) which had been pre-digested with AgeI and EcoRI. As a negative control, non-targeting control pLKO.1 (SHC002) vector from Sigma was used.

#### Luciferase assay

The assay is based on a dual luciferase reporter system, in which cells are transfected simultaneously with two vectors containing individual reporter enzymes. The vector in which the enhancer modules were cloned is defined “experimental-reporter” and its luciferase activity correlates with the function of the putative enhancer. The second vector is defined “control-reporter” and its luciferase activity is constitutive. This second vector is co-transfected with the experimental reporter to provide an internal control for transfection efficiency. The experimental-reporter vector used was the firefly luciferase pLS-mP-Luc (https://www.addgene.org/106253/). The control-reporter vector used was the Renilla luciferase pls-SV40-mP-Rluc (https://www.addgene.org/106292/) with constitutive expression of the reporter mediated by the SV40 enhancer. Each putative enhancer module was PCR amplified from Fujioka AML cell genomic DNA and cloned into the “experimental-reporter" digested with XbaI and SbfI. Each module was inserted in front of a minimal promoter regulating the expression of the luciferase reporter gene. As a negative control, part of the *ACTB* genomic locus was cloned. As a positive control, the sequence corresponding to the SV40 enhancer from pls-SV40-mP-Rluc vector, was cloned upstream of the minimal promoter in the pLS-mP-Luc vector. Oligonucleotides used for the PCR amplification are shown in Table S4.

The production of lentiviral particles containing luciferase reporter sequences was performed as described above. Specifically, each firefly luciferase plasmid was individually incorporated into lentivirus along with the control-reporter using a molecular ratio of 1:10. The production of lentiviral supernatants using HEK293 cells, and the transduction of leukemic human cells was performed as described above. Forty-eight hours after transfection, both firefly and Renilla luciferase activity, associated with the experimental-reporter and the control-reporter respectively, were analyzed with the Dual-Glo Luciferase Assay System (Promega) following the manufacturer’s instructions. The luciferase activity for each candidate enhancer was normalized to the respective Renilla activity.

#### Flow cytometry

Stanford Modified (SM) buffer was used as cell suspension media for incubation with antibodies for immunophenotypic analysis. SM buffer consisted of 479mL phenol red free RPMI 1640 medium (Sigma Aldrich) supplemented with 15mL (3%) FBS and 1mM EDTA (Fisher Scientific). Flow cytometry analyses were performed using an LSR Model II flow cytometer (BD Biosciences, Oxford, UK). For the differentiation assay anti-human CD86-PerCPeFluor710 antibody (Thermo Fisher) was used. Apoptosis was assessed using a BD Pharmingen APC Annexin V kit.

#### Cytospin analyses

2.5x10^4^ cells were resuspended in 150μL PBS and, through centrifugation at 60xg for 5 minutes, were spun onto a microscope glass slide and left to air dry. Cells were fixed by incubation in methanol for 10 minutes followed by May-Grunwald (Sigma; diluted 1:1 with Sorenson’s Buffer (33.3mM KH_2_PO_4_, 64.75mM Na_2_HPO_4_, pH6.8)) staining for 20 minutes and subsequent staining with Giemsa (Sigma; 10x diluted with Sorenson’s Buffer) for 30 minutes. Finally, stained cells were washed under running tap water and left in Sorenson’s buffer for five minutes prior to one final wash with tap water. Slides were left to air dry before cells were permanently mounted with a coverslip and DPX neutral mounting media (VWR, Radnor, PA). Images were obtained using a Leica SCN400 histology scanner (Leica, Solms, Germany) and analyzed using SlidePath Gateway software v1.0 (Leica).

#### Quantitative PCR

Total RNA was extracted using QIAshredder spin columns and an RNeasy Plus Micro kit or Mini kit (QIAGEN, Manchester, UK) as per manufacturer’s instructions. For quantitative PCR, purified RNA was reverse-transcribed to cDNA using the High-Capacity cDNA Reverse Transcription Kit following manufacturer’s instructions (Thermo Fisher). qPCR reactions were performed in triplicate from cDNA with a concentration between 1 and 25ng, and expression of the housekeeping gene *ACTB* was used for normalization. The qPCR assays were executed using 2x SYBR Green Mastermix (Thermo Fisher) in MicroAmp® optical 384-well reaction plates (Applied Biosystems) and analyzed using an Applied Biosystems 7900HT Sequence Detection System. Primers used are shown in Table S4. ΔCt values relative to *ACTB* were assessed using SDS software v2.1 (Applied Biosystems).

#### Evaluation of H3K27ac ChIP signal along *IRX3* TAD

H3K27Ac ChIP-seq data for AML primary samples were retrieved from McKeown et al.^14^ (www.ncbi.nlm.nih.gov/sra, accession number SRP103200). Details of each primary sample can be found in Table S1. Samples were divided into groups according to the expression value of *IRX3* and *FTO*-lncAML and for each sample H3K27Ac ChIP signal (number of mapped reads for each genomic bin of 1kb within the IRX3 TAD) was calculated.

#### Chromatin immunoprecipitation and next generation sequencing

ChIP in primary AML samples for histone mark H3K27ac or in Fujioka cells for histone mark H3K4me1 and MED1 was performed as previously described in Lee et al.^38^ For primary AML samples 1x10^7^ cells were used while for Fujioka cells 1x10^7^ and 1x10^8^ cells were used for H3K4me1 and MED1 respectively. Cells were cross-linked at room temperature using 1% formaldehyde. For MED1 ChIPseq a previous step of Gold cross-linking (Diagenode) was included, as per manufacturer’s instructions. After 10 minutes the reaction was quenched by incubation for 10 minutes with 0.125M glycine. Cell pellets were washed twice with cold PBS and 1x10^7^ or 1x10^8^ cells were used per ChIP. Briefly, nuclear lysates were sonicated using a Bioruptor Plus (Diagenode) for 6-10 min at high, 30s ON, 30s OFF settings for primary AML cells and for 8 min at high, 30s ON, 30s OFF settings for Fujioka cells. Immunoprecipitation was performed overnight at 20rpm and 4°C, with 10μl magnetic beads (Dynabeads (Protein G), Invitrogen, Carlsbad, CA) per 1μg antibody per 1x10^7^ cells. After washing five times with RIPA buffer (50mM HEPES pH 7.6, 1mM EDTA, 0.7% Na deoxycholate, 1% NP-40, 0.5M LiCl), chromatin IP-bound fractions were extracted at 65°C for 30min with elution buffer (50mM Tris-HCl pH 8.0, 10mM EDTA, 1% SDS) vortexing frequently. Cross-linking was then reversed by incubation at 65°C for 16 hours. RNAseA (1mg/ml) and proteinase K (20mg/ml) were used to eliminate any RNA or protein from the samples. Finally, DNA was extracted using phenol:chloroform:isoamyl alcohol extraction and precipitated with ethanol (adding two volumes of ice-cold 100% ethanol, glycogen (20μg/μl) and 200mM NaCl) for at least 1 hour at -80°C. Pellets were washed with 70% ethanol and eluted in 30μl 10mM Tris-HCl pH 8.0. Libraries were prepared for sequencing using a Microplex Library Preparation Kit (Diagenode) and 1ng ChIP to the manufacturer’s instructions. Fragments of 200–800 bp were selected using AMPure beads (Beckman Coulter, Pasadena, CA) and quantified by quantitative PCR with a KAPA Library Quantification Kit (Roche). ChIP-seq data were generated using the NextSeq500 desktop sequencing system (Illumina) with 2x75bp Hi Output.

Reads were aligned to the human genome (GRCh38 GENCODE release 25) using BWA-MEM version 0.7.15.^35^ Reads were filtered using Samtools (version 0.1.9)^39^ to keep only reads that mapped to standard chromosomes and Bedtools version 2.25.0^40^ to remove reads mapped to blacklisted regions defined by ENCODE (http://mitra.stanford.edu/kundaje). ChIP-seq data figures were made using the UCSC genome browser hg38^41^ (genome.ucsc.edu) and Adobe Illustrator.

#### ChIP-qPCR

Fujioka cells were infected with lentiviral particles targeting *FTO*-lncAML or a non-targeting control. Next day, cells were drug selected with puromycin 3μg/mL and incubated for two days. 1x10^7^ cells were used for ChIP with the antibody acetyl-H3K27. ChIP DNA was then used as a template for SYBR Green qPCR as described above. Ct values obtained for each region in the ChIP samples were normalized to the Ct values obtained for the same region in the input. Typically 1% of starting chromatin which was not subjected to IP was used as input. Eventually, the normalized Ct values were used to compare the enrichment of each region between control and *FTO*-lncAML KDs. Primers used are shown in Table S4.

#### MeDIP-seq

Genomic DNA for human methylated DNA immunoprecipitation (MeDIP) was isolated from AML patient blasts using the QIAamp DNA blood Mini Kit (QIAGEN). gDNA samples were diluted in GenDNA TE buffer (Diagenode) to reach a concentration of 100ng/μl and 100μl was then sheared in 0.65ml Bioruptor Microtubes (Diagenode). gDNA samples were sonicated for 13 min at high, 30 sec ON, 30 sec OFF settings to 200–300 bp using Diagenode’s Bioruptor platform and the size distribution was confirmed through gel electrophoresis. Barcoded adaptors for Illumina sequencing were added to 1μg of fragmented genomic DNA per experiment, using the NEB Next Ultra II DNA Library Prep Kit for Illumina (New England Biolabs), following the manufacturer’s instructions. This protocol was stopped after end repairing, adaptor ligation and clean-up (and before any amplification steps), and the adaptor ligated fragmented DNA was then used for hydroxymethylated DNA pulldown. This was performed using the MagMeDIP kit (Diagenode) following manufacturer’s instructions. Briefly, magnetic beads were washed twice with ice cold bead wash buffer and incubated overnight with the denatured adaptor-ligated DNA and the 5-methylcytosine monoclonal antibody 33D3 (C15200081, Diagenode). The day after, DNA IP samples were washed three times in ice-cold MagWash Buffer-1 and once in ice-cold MagWash Buffer-2 and DNA was isolated using the iPure kit v2 (C03010014, Diagenode) following the manufacturer’s instructions. Purified methylated genomic DNA was then further processed with a NEB Next Ultra II DNA Library Prep Kit for Illumina at the step of PCR enrichment of adaptor-ligated DNA, per the manufacturer’s instructions, continuing with the entire protocol to prepare libraries for Illumina sequencing. Before PCR amplification, DNA concentration was calculated using the Qubit dsDNA HS Assay kit (Life Technologies) to determine the number of cycles required for PCR enrichment. Libraries were then barcoded, pooled and sequenced using a NextSeq500 desktop sequencing system (Illumina) with 75bp, paired-end high output generating 73–90 million reads per sample. Reads were aligned to the human genome (hg38) using BWA-MEM version 0.7.15.^35^ MEDIPS^36^ software was used to calculate counts, RPKM (reads per kilobase of transcript per million mapped reads) and RMS (raw methylation score) values and to perform differential methylated region analysis. MeDIP data figures were made using the UCSC genome browser hg38^41^ (genome.ucsc.edu) and Adobe Illustrator.

#### 4Cseq

4Cseq library preparation for Fujioka cells, primary samples and Fujioka cells after *FTO*-lncAML KD was performed as previously described.^42^ In brief, a total of 1x10^7^cells was resuspended in 10ml of PBS/10%FBS and crosslinked with formaldehyde at a final concentration of 2%. The resuspended cells were incubated for 10 minutes at room temperature while tumbling and subsequently the formaldehyde activity was quenched using glycine at a final concentration of 0.125M. The quenched solution was then lysed in 1ml of lysis buffer (50mM Tris–HCl at pH 7.5, 150mM NaCl, 5mM EDTA, 0.5% NP-40, 1% TX-100 and 1x complete protease inhibitors, Roche). Isolated nuclei were resuspended in 450μl of Milli-Q water and digested with a 4bp cutter restriction enzyme (DpnII) (NEB). After the first digestion step, samples were subjected to proximity ligation overnight at 16°C using 50U of T4 DNA Ligase (Sigma-Aldrich). The ligated chromatin was then de-cross-linked overnight with 30μl of Protein K (10mg/ml) and DNA was purified using phenol-chloroform and subjected to a second digestion with the 4bp cutter restriction enzyme (NlaIII) (NEB). Digested DNA was then subjected to a second step of proximity ligation overnight and eventually DNA was extracted using phenol-chloroform and further purified using the QIAquick PCR purification kit (QIAGEN). The resulting 4Cseq libraries were amplified to generate fragments containing the viewpoint ligation partners. Each primer contained specific Illumina sequencing adaptors which allow the amplified DNA fragments to adhere to the flow-cell where the sequencing takes place. PCR primers were PAGE purified from IDT and contained the P5/P7 Illumina adapter sequence, a barcode for sample identification and the specific viewpoint restriction enzyme sequence. The set of primers was designed using the 4Cseq primer design tool of the University of Chicago (https://mnlab.uchicago.edu/4Cpd/). The primers used to amplify both Fujioka and primary samples libraries were (5’-3’):

Reading-primer:

aatgatacggcgaccaccgagatctacactctttccctacacgacgctcttccgatctgaaggtgggcgcccgatc.

Non-reading primer:

caagcagaagacggcatacgagatgtgactctgccgtggagtg.

PCR reactions were performed with the Expand Long Template polymerase (Roche) using 3.2μg of 4C template product using the following thermocycling conditions: 94°C for 2min, 30 cycles of (94°C for 10s, 55°C for 1min, 68°C for 3min), 68°C for 5min. Subsequently, the 16 reactions were pooled together and purified using the High Pure PCR Product Purification Kit (Roche). At this point, the libraries were quantified using the Nanodrop spectrophotometer and quality was assessed by running 300ng of each library on a 1.5% agarose gel. The obtained libraries were quality checked using the Agilent 2100 Bioanalyzer (Agilent technologies, Santa Clara, CA) and subsequently sequenced with 10% phiX using a MiSeq desktop sequencing system (Illumina) with a setting of 76-bp single-end. The sequencing generated around 1 million reads for Fujioka cells, primary samples and around 3 million reads for Fujioka cells after *FTO*-lncAML KD. Sequencing data was deconvoluted using Cutadapt version 1.18. Reads were mapped and analysis performed using 4Cseq pipe,^43^ 4CSeq,^44^ and 4C-ker^45^ software.

#### Rapid amplification of cDNA ends (RACE)

3′ and 5′ cDNA ends characterization, was performed using the Smarter RACE kit (Takara Bio). In brief, single-strand cDNA was synthesized from 1μg of Fujioka cells RNA using 5′ and 3′ RACE CDS primers with poly-T, and SMARTer IIA oligo for template switching for the 5′ amplification. The following step of 5′ and 3′ amplification was performed through a touchdown PCR using the Universal Primer A coupled with a specific 5′ or 3′ primer. The 5′ and 3′ primers were (5′-3′):

5′ specific primer:

gattacgccaagcttcgggcaattcgtgactggcaccttaaaagc.

3′ specific primer:

gattacgccaagcttgcgtagctatatcggggatccaaaggtt.

The gene specific primers were designed to include a 5′-tail of nucleotides overlapping sequence of the pRace vector to allow the subsequent step of cloning. PCRs for both the 3′ and 5′ amplification were set up with the following thermal cycling conditions: 5 cycles of (94°C for 30s, 72°C for 3min), 5 cycles of (94°C for 30s, 70°C for 30s, 72°C for 3min) and 25 cycles of (94°C for 30s, 68°C for 30s, 72°C for 3min). Afterwards PCR products were separated on agarose gel and then purified using the NucleoSpin Gel and PCR Clean-Up Kit (Takara Bio) following the manufacturer’s instructions, and eventually cloned into the pRace vector using the In-Fusion HD Cloning Kit (Takara Bio). Specifically, 7μl of the gel-purified RACE product were combined with 1μl of the linearized pRACE vector and 2μl of the In-fusion cloning Master Mix. The solution was then incubated for 15 minutes at 50°C and eventually transformed into Stellar Competent Cells (Takara Bio) according to the manufacturer’s instructions. The following day, colonies for each 5′ and 3′ amplification were individually picked, and plasmid DNA was extracted using standard miniprep methods. Eventually, the plasmid DNA for each colony was sequenced by the Sanger method. RACE figures were made using the BLAT (BLAST-Like Alignment Tool) function included in the UCSC genome browser hg38 and paint3D.

#### CRISPR Cas9 mediated deletion of enhancers

The deletion of each enhancer module and the control region was performed through electroporation in Fujioka cells of a ribonucleoprotein (RNP) complex consisting of Cas9 protein and a total of four guide RNAs for each enhancer. The guides were designed using the tool CRISPOR (http://crispor.tefor.net/) and are shown in Table S4.

Guides were produced by Synthego (www.synthego.com/) and resuspended in TE buffer to a concentration of 50μM. For the constitution of the RNP complex, the four guides were mixed in a 1:1:1:1 ratio to a concentration of 44μM in a volume of 0.5μl. This was mixed in a 1:1 ratio with the Alt-R Cas9 previously diluted to 36uM in electroporation buffer R included with the Neon Transfection Kit (Thermo Fisher). The complex was then incubated for 20 minutes at RT. As a non-targeting control, an RNP complex was constituted using a scrambled guide resuspended to 50μM and diluted to 0.44μM in a volume of 0.5μl and again mixed in a 1:1 ratio with the Alt-R. Electroporation was performed using the Neon Transfection System. For each condition, a total of 2 million cells was electroporated in 10 consecutive reactions of 200,000 cells each. Specifically, cells were washed once in PBS and resuspended in 10μL buffer R, for a total of 100μl per condition. Afterwards, 1μl of the RNP complex was added to the 10μl cells and the mixture was incubated at room temperature for 5 minutes. Cells were eventually electroporated using 10μl tips and transferred immediately in culture. After 48 hours, a sub-fraction of cells was used to extract genomic DNA using a DNeasy Blood and Tissue kit (Qiagen) in order to confirm the deletion through PCR amplification of each enhancer module.

Primers used for DNA amplification are shown in Table S4. The remaining cells were used to extract RNA for qPCR analysis.

#### RNA sequencing library preparation

Total RNA was extracted from 2x10^6^ Fujioka cells or 5x10^5^ AML primary AML blast cells using the RNeasy Plus Mini kit (QIAGEN) and the RNeasy Plus Micro kit (QIAGEN) respectively. Samples were homogenized using QIAshredder spin columns while genomic DNA was removed by using gDNA eliminator columns. Additionally, extracted RNA from primary samples was DNAse treated using the TURBO DNA-*free* Kit (Thermo Fisher) according to the manufacturer’s instructions. Before sequencing, RNA quality was evaluated using a 2100 Bioanalyzer (Agilent Technologies). RNAseq libraries were prepared using the SureSelect PolyA (Agilent Technologies) according to the manufacturer’s instructions for Fujioka cells, while the TruSeq Stranded Total RNA kit (Illumina) was used for AML primary samples. The TruSeq kit includes a ribo-depletion step to eliminate ribosomal RNAs and it captures coding RNA plus multiple forms of non-coding RNA. All libraries were quantified by quantitative PCR using a Kapa Library Quantification Kit for Illumina sequencing platforms (Roche). Libraries were then barcoded, pooled and sequenced using Novaseq6000 (Illumina) or a NextSeq500 desktop sequencing system (Illumina) for primary samples. A single run (400 million reads) produced a mean of 56.6-68.2 million 100 bp paired-end reads in the case of SureSelect –polyA or 49.4-54.72 million 75 bp paired-end reads in the case of TruSeq libraries.

#### *In silico* identification of *FTO*-lncAML transcripts and quantification of its abundance in primary samples

To characterize *in silico FTO*-lncAML transcripts, RNAseq reads from the 8 AML samples were aligned to the human genome (GRCh38 GENCODE release 25) using STAR version 2.7.9a with the parameter --outFilterMultimapNmax 1 ,--outFilterType BySJout, --alignSJoverhangMin 8.^16^ Next, the generated alignment files were used to generate single transcriptome for each of the primary sample using Cufflinks v2.2.1.^17^ Eventually, the tool Cuffmerge from Cufflinks v2.2.1 was used to merge the different assembled transcriptomes from each sample, and this resulted in a global new transcriptome which included the Cufflinks *in silico* predicted transcripts (Figure 3B). To quantify the expression of the *FTO*-lncAML transcripts in AML we used Kallisto^20^ using as an index the GTF file corresponding to hg19_ncbi_build37.2 and the sequence corresponding to the *in silico* predicted *FTO*-lncAML1. Transcripts per million (TPM) value of *FTO*-lncAML and the other genes were calculated. Additional data for analysis of FTO-lncAML expression in normal CD34^+^ HSPCs were downloaded from Assi et al.^46^

#### Data analysis for RNA sequencing of Fujioka cells after *FTO*-lncAML and *IRX3* KD

Reads were aligned to the human genome (GRCh38 GENCODE release 25) and genes were annotated using the corresponding GTF file (GENCODE GRCh38 release 25) using STAR version 2.4.2a with the parameter --outFilterMultimapNmax 20 ,--outFilterType BySJout, --alignSJoverhangMin 8, --quantMode GeneCounts.^16^ To perform the differential gene expression analysis between NTCs and KDs DEseq2 was used. FPKM (fragments per kilobase of transcript per million mapped reads) values for each gene were calculated.^37^

#### Coding potential assessment

FASTA sequences for *FTO-*206-AML, *FTO*-lncAML1, *FTO*-lncAML2, GAPDH (ENST00000229239.10) and U6 (ENST00000613107.1) were individually submitted to the CPC2 (coding potential calculator) pipeline.^47^ The coding probability score for each transcript was determined and sequences with a value below 0.5 were considered non-coding.

#### Subcellular fractionation for detection of RNAs through qPCR

Fractionation was performed using a two-step differential centrifugation protocol as described in Conrad and Ørom.^48^ Briefly, cells were initially lysed with a mild detergent (10mM Tris pH7.4, 150mM NaCl, 0.15% Igepal CA-630) to release the cellular constituents while leaving the nuclei intact. Afterwards, the lysate was centrifuged through a 24% sucrose solution which separates the nuclei (pellet) from the cytosol (supernatant). Eventually, RNA was extracted from each fraction with a standard Trizol-based procedure, and Ct values for *GAPDH*, *U6* small nuclear RNA and *FTO*-lncAML were evaluated using SYBR Green qPCR as described above. The proportion of each transcript in cytoplasm and nuclear compartments was calculated as:

$$Proportion\;of\;expression=\frac{2^{-Ct\left(compartment\right)}}{2^{-Ct\left(cytoplasm\right)}+2^{-Ct\left(nucleus\right)}}$$

Primers used are listed in Table S4.

### Quantification and statistical analysis

#### Gene Set Enrichment Analysis

Gene Set Enrichment Analyses (GSEA) were performed using the Broad Institute’s GSEA software GSEA v2.0.14 from www.broadinstitute.org/gsea. Gene sets are shown in Table S5 and are from the Molecular Signatures Database^49^ or are generated from data from Wouters et al.^50^ For the latter, genes “UP in FAB M4 M5 vs M1” are those expressed on average at least three-fold higher in 188 cases of French-American-British M4 and M5 versus 95 cases of M1 subtype and with *P*<0.001 by T-test. Genes “UP in FAB M1 vs M4 M5” are those expressed on average at least two-fold higher in M1 versus M4 and M5 cases, and with *P*<0.001 by T-test.

#### Statistics

Statistical analyses were performed using Microsoft Excel or GraphPad Prism software. Details of the statistical tests used for each analysis shown may be found in figure legends.

## Data Availability

• ChIP-seq and RNA-seq data have been deposited at GEO and are publicly available as of the date of publication. Accession numbers are listed in the key resources table. • This paper does not report original code. • Any additional information required to reanalyze the data reported in this paper is available from the lead contact on request.

## Supplemental information

Document S1. Figures S1–S6

Table S1. Karyotypes, mutational profiles, IRX3 expression and FTO-lncAML expression of primary AML samples Samples with BB numbers (blue cells) are from the Manchester Cancer Research Centre Tissue Biobank. Samples with SA numbers (yellow cells) are from McKeown et al.^14^ related to Figures 1–3.

Table S2. Genes differentially expressed after FTO-lncAML or IRX3 knockdown, related to Figure 7

Table S3. Genes differentially expressed in FTO-lncRNA^high^, IRX3^high^ versus FTO-lncRNA^low^, IRX3^high^ primary AML cases (data from McKeown et al.^14^), related to Figure 7

Table S4. Oligonucleotide sequences, related to STAR Methods

Table S5. Gene sets, related to STAR Methods
