# Supplementary material for: Differentiation block in acute myeloid leukemia regulated by intronic sequences of FTO
Source: iScience. 2023 Jul 11;26(8):107319. doi: 10.1016/j.isci.2023.107319 (PMC10393733; doi:10.1016/j.isci.2023.107319)
Supplement: Document S1. Figures S1–S6 [file mmc1.pdf]

iScience, Volume *26*

Supplemental information

Differentiation block in acute myeloid leukemia regulated by intronic sequences of *FTO*

Francesco Camera, Isabel Romero-Camarero, Bradley H. Revell, Fabio M.R. Amaral, Oliver J. Sinclair, Fabrizio Simeoni, Daniel H. Wiseman, Lovorka Stojic, and Tim C.P. Somervaille

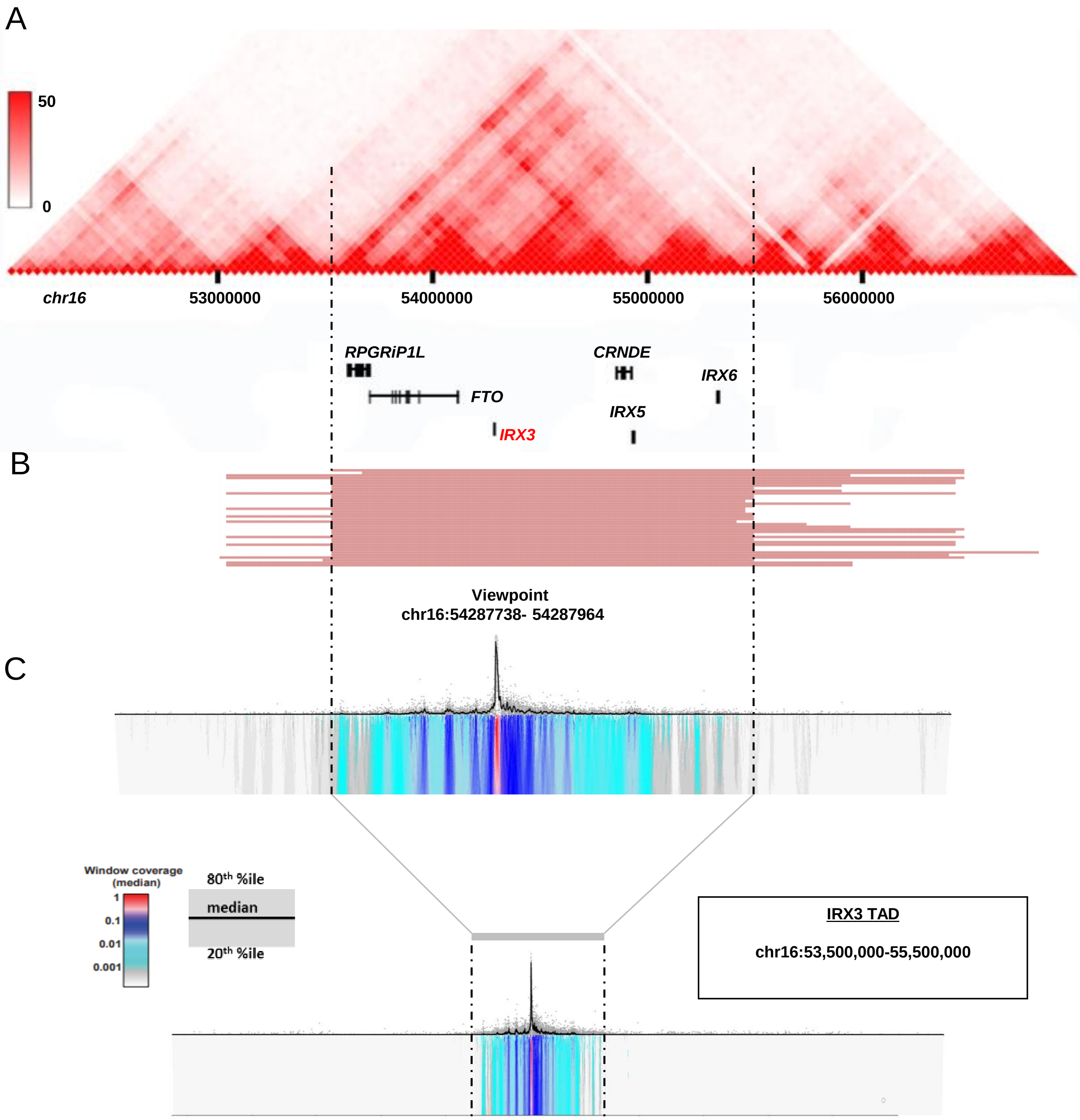

Figure S1. Characterization of the *IRX3* topologically associating domain, related to Figure 1.

(A) Heat map shows chromatin conformation analysis in THP1 cells as determined by Hi-C (Wang et al., 2018). The region shown surrounds the *IRX3* locus. (B) Graphical representation of *IRX3* TADs from a cohort of human tissue or cell lines. Each horizontal line represents the chromosomal coordinates of the *IRX3* TAD from a different cell line or tissue (data from 3dgenome.fsm.northwestern.edu). (C) 4C-seq interaction diagram for *IRX3* promoter in Fujioka cells with two different resolutions. In these plots, the line graph represents the contact intensity trend depicted by the median of normalized coverage for running windows of size 5kb and surrounded by the 20th and 80th percentile trends. The heat map represents the enrichment relative to the maximum attainable 12kb median value calculated for sliding windows (2-50kb) of linearly increasing size as a gradient from red (highest interaction) to blue to cyan to white (lowest interaction). Vertical dashed lines indicate *IRX3* TAD boundaries as defined from the Hi-C analysis and 4C-seq.

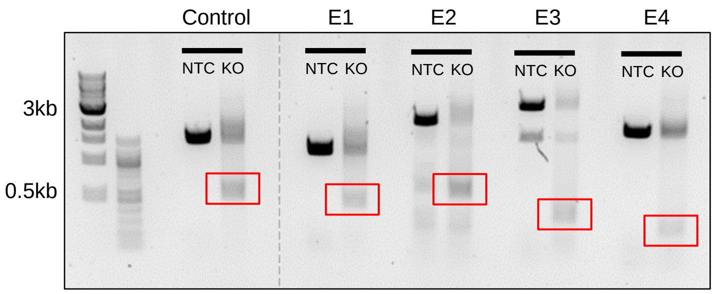

Figure S2. CRISPR deletion, related to Figure 2.

PCR amplification of each of the indicated control or enhancer regions shows deletion of the targeted enhancer in each of the knockout (KO) conditions. Red boxes indicate the PCR product expected from each deleted enhancer. Vertical dotted line indicates removal of excess gel space between relevant lanes.

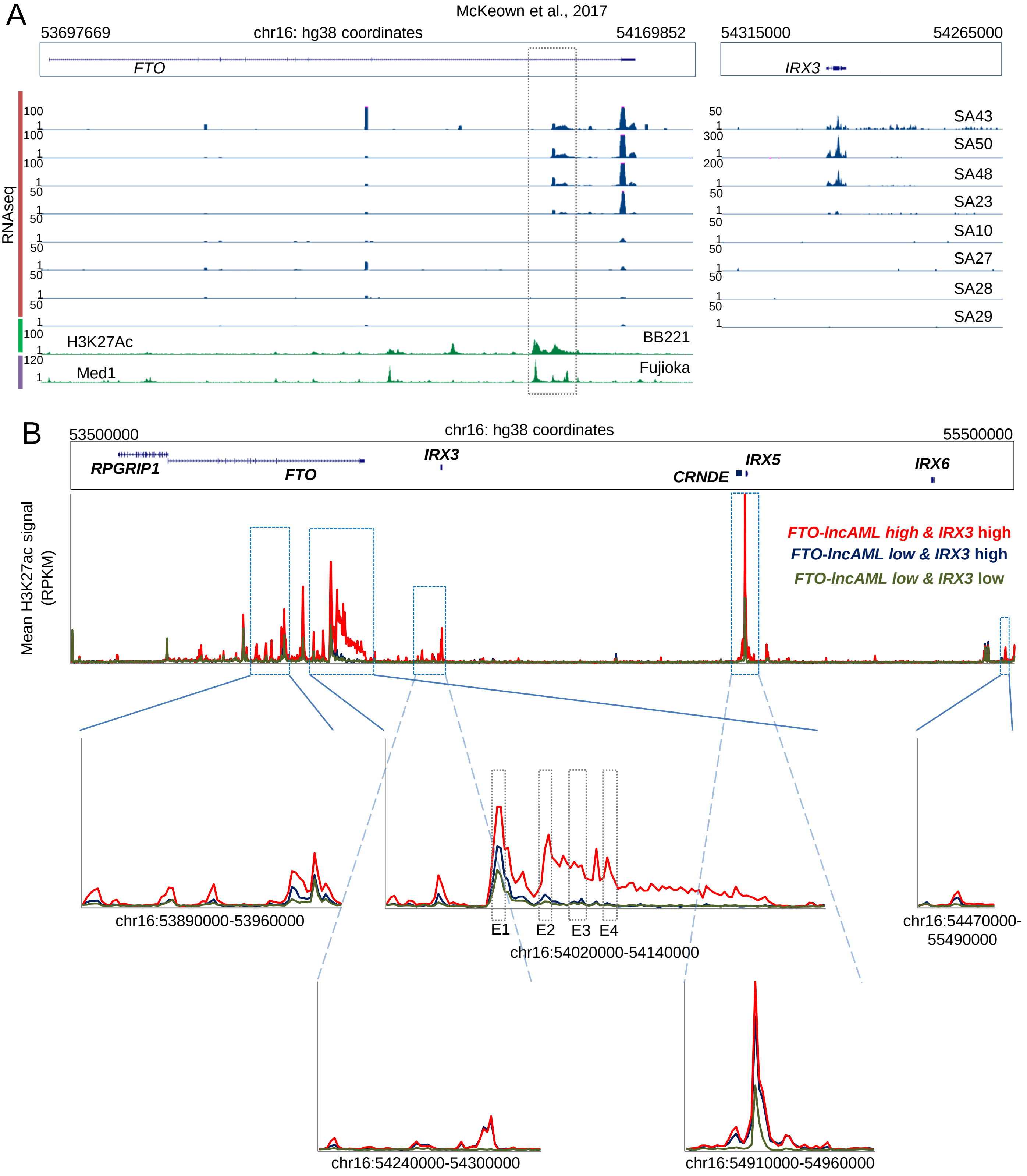

Figure S3. Unannotated transcripts arising from intron 8 of *FTO* in AML samples, related to Figure 3.

(A) RNAseq tracks surrounding the terminal part of *FTO* (left) and *IRX3* (right) in *IRX3*^{high} and *IRX3*^{low} primary (data derived from McKeown et al., 2017). ChIPseq tracks for H3K27ac and MED1 are also shown. (B) Graph shows mean H3K27ac ChIP signal over the *IRX3* TAD for primary samples grouped according to the indicated categories: *FTO*-lncAML^{high} *IRX3*^{high} (red), *FTO*-lncAML^{low} *IRX3*^{high} (blue) and *FTO*-lncAML^{low} *IRX3*^{low} (green). Data are derived from McKeown et al., 2017. Areas with significant differential acetylation are highlighted by dashed lines and enlarged below.

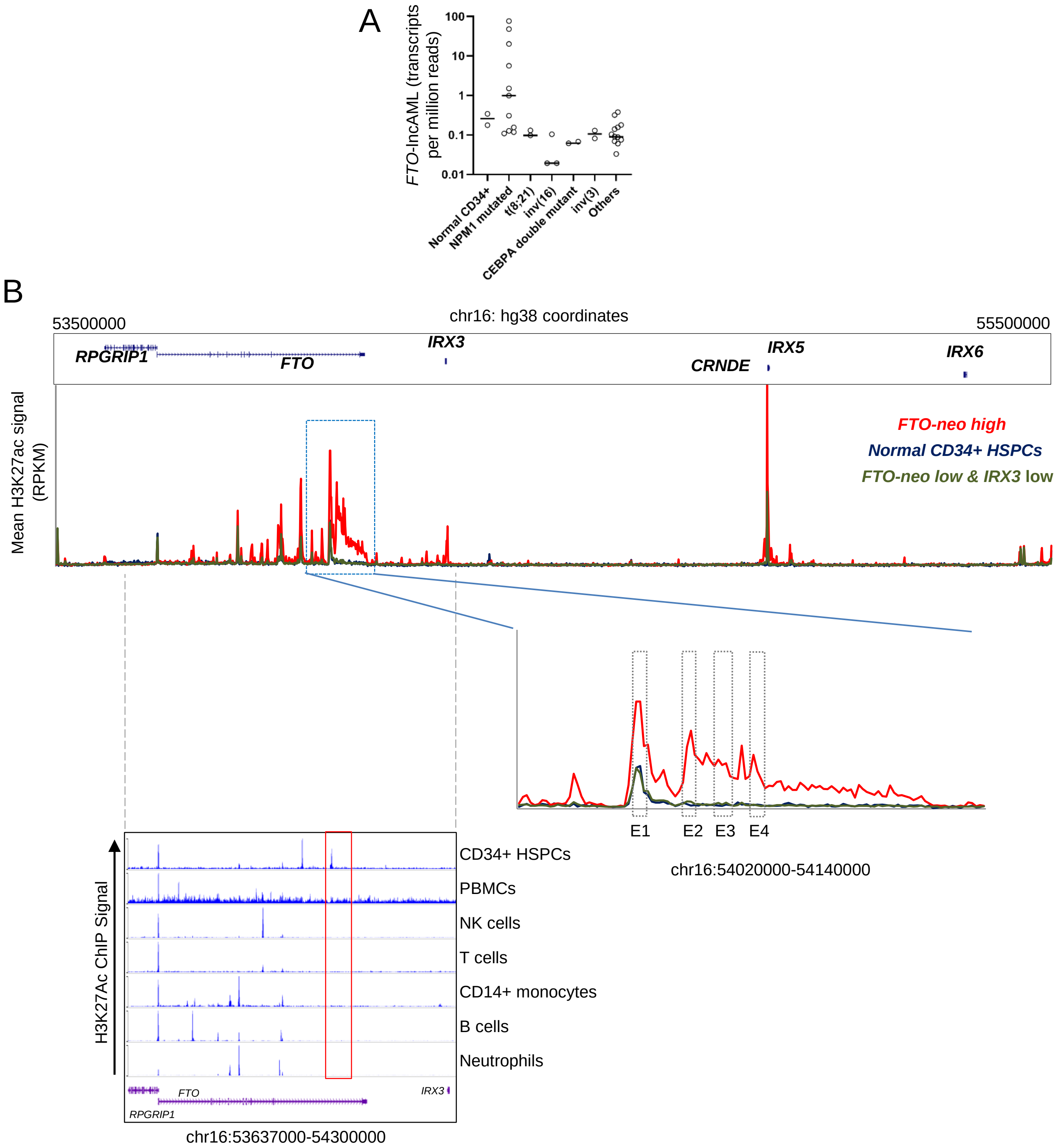

Figure S4. Expression of *FTO*-lncAML and epigenetic marking of intron 8 of FTO in normal hematopoiesis, related to Figure 3.

(A) Expression of *FTO*-lncAML in normal human CD34$^+$ stem and progenitor cells (n=2) and primary human AML samples (n=36) from Assi et al.[37] (B) Graph shows mean H3K27ac ChIP signal over the *IRX3* TAD for primary samples grouped according to the indicated categories. Data are derived from McKeown et al., 2017. The genomic area corresponding to the region containing E1-E4 is highlighted by dashed lines and enlarged below and to the right. Below and to the left are shown ChIPseq tracks for H3K27ac over the FTO-IRX3 genomic locus in the indicated normal human cell types. Data are derived from ENCODE and the WashU Epigenome Browser (ENCODE Project Consortium, 2012; Li et al., 2022). HSPC, hematopoietic stem and progenitor cells; PBMC, peripheral blood mononuclear cells; NK, natural killer.

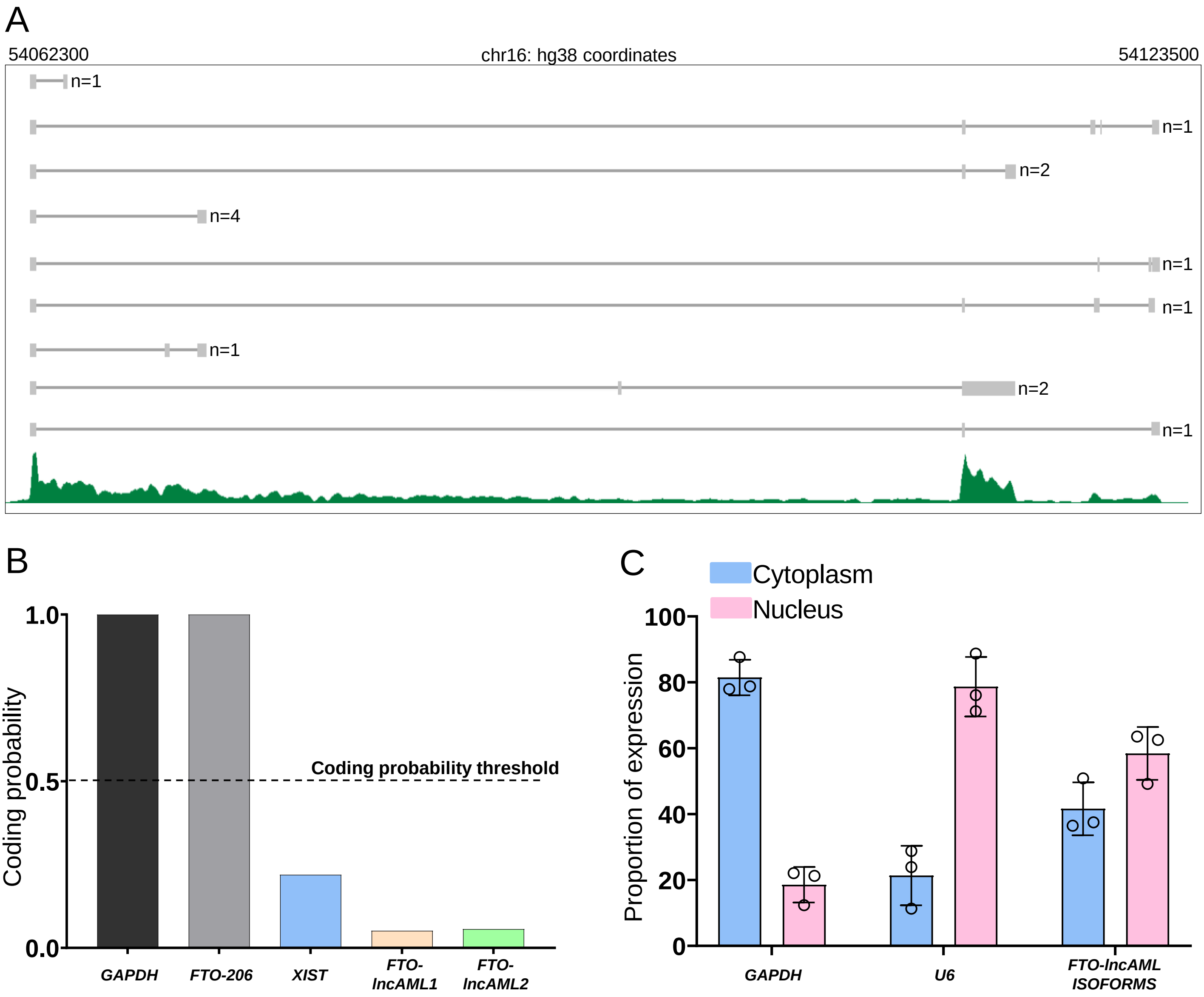

Figure S5. Characterization of *FTO-lncAML* transcripts, related to Figure 4.

(A) Representation of 3'RACE sequencing results for clones which did not terminate at the end of *FTO*-lncAML exon 2 (shared with the last exon of *FTO*-206). Sequences are shown alongside the RNAseq track from primary AML sample BB221. (B) Coding probability scores for the two isoforms of *FTO-l*ncAML and *FTO*-206-AML as calculated by CPC2 (Coding Potential Calculator). *GAPDH* was used as positive control for a protein coding gene, while *XIST* was used as positive control for a non-coding gene. The accepted threshold for coding potential (0.5) is indicated. (C) Relative enrichment of the indicated transcripts in the indicated sub-cellular fractions in Fujioka AML cells, as determined by qPCR. Mean±SEM of three biological replicates.

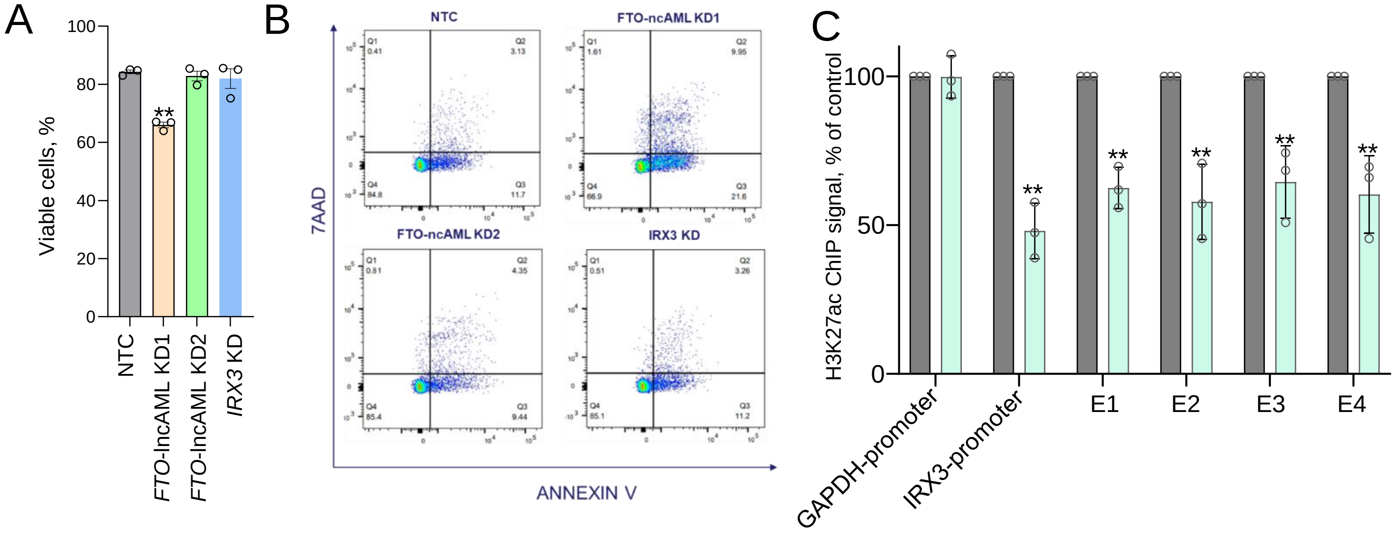

Figure S6. Consequences of *FTO*-lncAML knockdown, related to Figure 5.

(A) Bar chart shows mean+SEM percentage of viable cells as determined by Annexin V/7AAD analysis seven days following initiation of KD (n=3). ** indicates p<0.01 for the indicated comparisons by one way ANOVA with Tukey post hoc test. (B) Representative flow cytometry plots. (C) ChIP-qPCR for H3K27ac in Fujioka cells after *FTO*-lncAML KD. Data are normalized to amplification in the INPUT and showed as fold change relative to the NTC. Mean±SEM, n=3. ** indicates p<0.01 for the comparison of ChIP signal at E1-E4 and the IRX3 promoter versus GAPDH promoter, by one way ANOVA with Tukey post hoc test.
